# A Dynamic and Responsive Host in Action: Light‐Controlled Molecular Encapsulation

**DOI:** 10.1002/anie.201607693

**Published:** 2016-10-28

**Authors:** Seán T. J. Ryan, Jesús del Barrio, Reynier Suardíaz, Daniel F. Ryan, Edina Rosta, Oren A. Scherman

**Affiliations:** ^1^ Melville Laboratory for Polymer Synthesis, Department of ChemistryUniversity of CambridgeLensfield RoadCambridgeCB2 1EWUK; ^2^ Schlumberger Gould ResearchMadingley RoadCambridgeCB3 0ELUK; ^3^ Melville Laboratory for Polymer Synthesis, Department of ChemistryUniversity of CambridgeLensfield RoadCambridgeCB2 1EWUK; ^4^ NASA Goddard Space Flight CenterGreenbeltMDUSA; ^5^ Department of ChemistryKing's College LondonLondonSE1 1DBUK; ^6^ Department of ChemistryKing's College LondonLondonSE1 1DBUK; ^7^ Melville Laboratory for Polymer Synthesis, Department of ChemistryUniversity of CambridgeLensfield RoadCambridgeCB2 1EWUK

**Keywords:** host–guest systems, hydrogen bonds, macrocycles, photochemistry, supramolecular chemistry

## Abstract

The rational design of a flexible molecular box, **oAzoBox** ^4+^, incoporating both photochromic and supramolecular recognition motifs is described. We exploit the *E*↔*Z* photoisomerization properties of azobenzenes to alter the shape of the cavity of the macrocycle upon absorption of light. Imidazolium motifs are used as hydrogen‐bonding donor components, allowing for sequestration of small molecule guests in acetonitrile. Upon *E*→*Z* photoisomerization of **oAzoBox** ^4+^ the guest is expelled from the macrocyclic cavity.

The encapsulation and active release of molecular species comprises an area of research that attracts constant attention and crosses both academic and industrial research interests, as encapsulation processes are ubiquitous in product synthesis and formulation.^1^, ^2^ One particular encapsulation strategy consists of the selective inclusion of guest compounds within the cavities of discrete, shape‐persistent macrocycles and has been applied to the solubilization and/or stabilization of active ingredients and hazardous materials, sensing, separation, and purification technologies.^3^, ^4^, ^5^, ^6^, ^7^, ^8^, ^9^ Owing to their dynamic nature, binding events in host–guest complexes can be controlled by a range of different stimuli. However, in spite of the many examples of noncovalent complexation, our ability to alter the interaction between a host and its guests is usually limited to invasive actions, such as the addition of strongly competing guest compounds or pH/redox switching.^10^, ^11^ Ideal triggering mechanisms should enable remote control over guest uptake and release in a well‐defined spatiotemporal fashion by a practical and easily operated stimulus, such as light. Indeed, the concept of photocontrolled uptake and release of guest species has been achieved by exploiting light‐responsive guests and, less frequently, host species.^12^, ^13^, ^14^

A few groups have provided examples of both strategies by controlling the dynamic encapsulation properties of cyclodextrines, calixarenes, cucurbiturils, metal–organic cages, and other systems with molecular switches, either appended to the host or as a guest molecule.^15^, ^16^, ^17^, ^18^, ^19^, ^20^, ^21^, ^22^, ^23^, ^24^, ^25^, ^26^ Systems which rely on the rearrangement or isomerization of a guest compound have a relatively limited scope of applicability. In our view, light‐switchable molecular containers may impact a much wider spectrum of technological applications. However, they also suffer from undesirable drawbacks, such as cumbersome synthesis and hindered isomerization properties by ring strain and molecular crowding.^27^

We report, here, the synthesis of **oAzoBox** ^4+^ (Figure 1), a photoresponsive molecular box produced by a facile three‐step synthetic procedure. We have made use of two *o*‐xylene‐bridged bis(imidazolium)‐azobenzene motifs to impart both light‐responsiveness and receptor‐like^28^, ^29^ properties to our macrocycle. In contrast to the rigid structure of more conventional azobenzene‐containing macrocycles,^27^, ^30^ **oAzoBox** ^4+^ exhibits a large and flexible architecture, which has two consequences: Firstly, the photochromic properties of **oAzoBox** ^4+^ are largely unaffected in comparison to model compound, **AzoBI** ^2+^ (Figure 1), by the embedment of the photoswitches in a cyclic architecture and secondly, its high flexibility is not detrimental to its recognition abilities. Therefore, **oAzoBox** ^4+^ is ideal for the realization of light‐controlled catch‐and‐release in solution.

**Figure 1:**
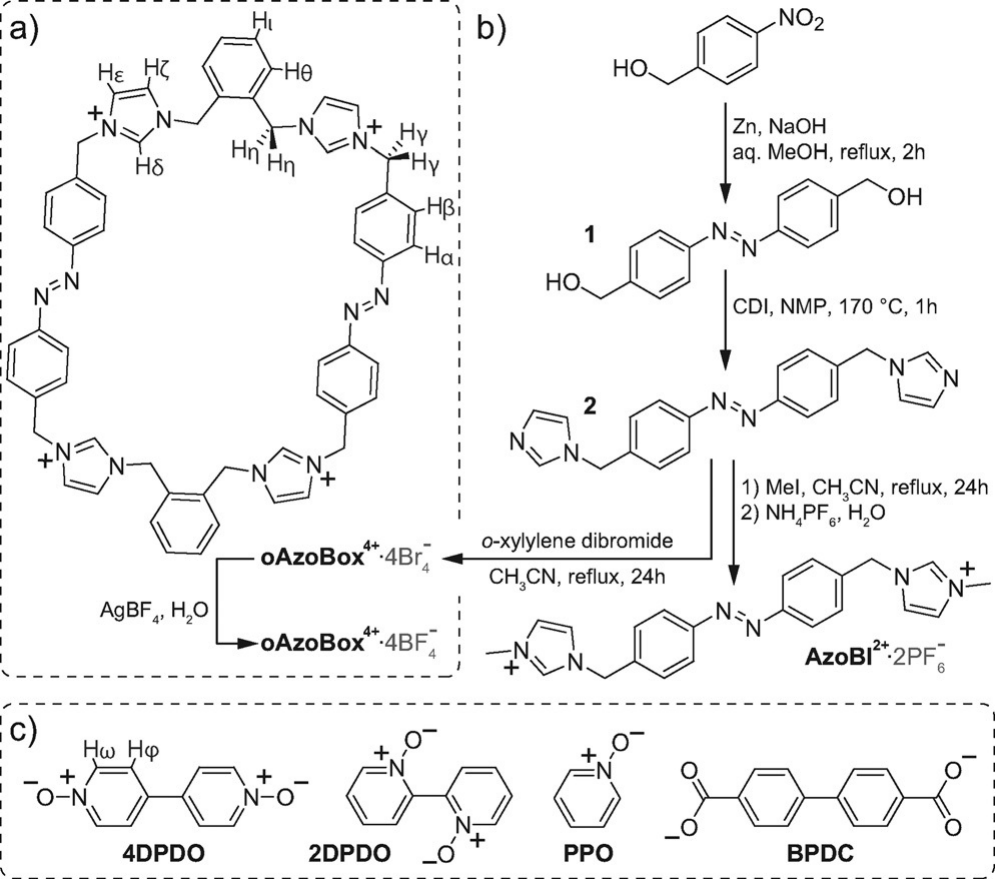
Chemical structure of **oAzoBox** ^4+^ (a), its synthesis (b), and small molecule guests used in this study (c).

**oAzoBox** ^4+^ was synthesized according to Figure 1 b. Firstly, the reduction of commercially available 4‐nitrobenzyl alcohol and subsequent reaction with CDI yielded intermediate **2**. Cyclization of **2** with an equimolar amount of *α*,*α*′‐dibromo‐*o*‐xylene, followed by salt metathesis, afforded **oAzoBox** *⋅* **4BF**_4_ in approximately 25 % yield after purification by recrystallization. The solid‐state structure (Figure 2) reveals that **oAzoBox** ^4+^ is substantially elongated, with a length of 21.05 Å, as measured by the distance between the centroids of the *o*‐xylene bridges. The breadth of the box, measured as the average distance between the planes of the two sets of parallel azobenzene phenyl units, was found as 4.3 Å, affording an aspect ratio of approximately 5.

**Figure 2:**
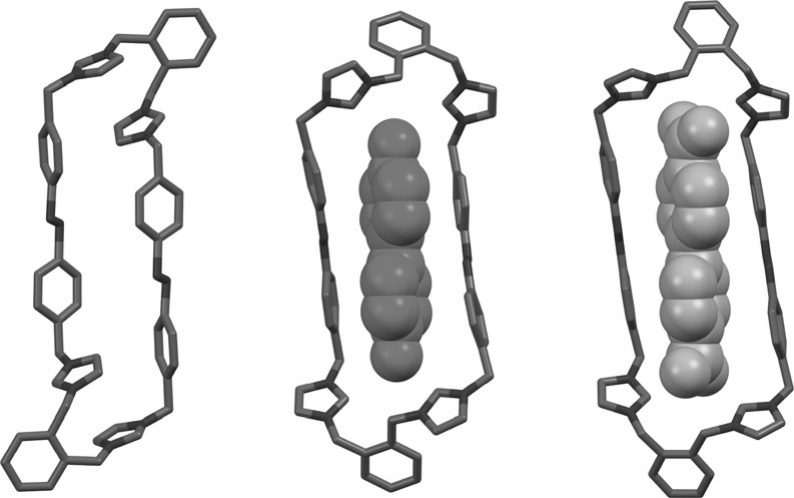
X‐ray crystal structure of *E*,*E*‐**oAzoBox** ^4+^ (left) and geometry‐optimized molecular structures (B3LYP‐D3(BJ)/TZVP) of *E*,*E*‐**oAzoBox** ^4+^⊂**4DPDO** (middle) and *E*,*E*‐**oAzoBox** ^4+^⊂**BPDC** (right).

The photoisomerization properties of model azobenzene, **AzoBI** ^2+^, were first examined by electronic absorption spectroscopy. **AzoBI** ^2+^ (see Figure S1 in the Supporting Information) exhibits a characteristic strong π–π* absorption band at short wavelengths (*λ*_max_=321 nm) and a weaker n–π* absorption band at longer wavelengths (*λ*_max_=445 nm). Upon UV light irradiation (350 nm), the intensity of the band corresponding to the π–π* transition strongly decreased, whereas that of the n–π* transition slightly increased. These spectroscopic changes can be directly ascribed to the *E*→*Z* photoisomerization, which can be reverted using visible light (420 nm). The electronic absorption spectrum of **oAzoBox** ^4+^ matches that of **AzoBI** ^2+^. The spectral changes associated to the *E*→*Z* photoisomerization of **oAzoBox** ^4+^ are analogous to those of **AzoBI** ^2+^. Therefore, it was estimated that the *E*↔*Z* photoisomerization behaviors of **AzoBI** ^2+^ and **oAzoBox** ^4+^ should closely resemble one another.

The ^1^H NMR spectrum of a freshly prepared solution of **oAzoBox** ^4+^ (Figure 3 c) shows nine distinct resonances, which are consistent with the *all‐trans E*,*E*‐**oAzoBox** ^4+^ stereoisomer. When a solution of *E*,*E*‐**oAzoBox** ^4+^ is irradiated with UV light, a new set of signals arises, which evidences the generation of an isomeric mixture of multiple components. The new aliphatic signals in the 5.00–5.50 ppm region are shifted upfield relative to the H*η* proton resonance of *E*,*E*‐**oAzoBox** ^4+^, which is consistent with the changes associated to the *E*→*Z* **AzoBI** ^2+^ photoisomerization. Furthermore, the intensity of the H*α* proton resonance of *E*,*E*‐**oAzoBox** ^4+^ decreases and three additional sharp and well‐resolved resonances, which are associated to the same type of H*α* resonance, appear at 7.95, 6.88, and 6.75 ppm. In combination, these results suggest that the UV‐light‐promoted *E*→*Z* isomerization generates a mixture of three distinct stereoisomers which, at the photostationary state, can be identified as *E*,*E*‐**oAzoBox** ^4+^ (18 %), *E*,*Z*‐**oAzoBox** ^4+^ (38 %), and *Z*,*Z*‐**oAzoBox** ^4+^ (44 %). The complete assignment of all proton resonances of each individual stereoisomer was achieved using two‐dimensional COSY and ROESY ^1^H NMR spectroscopy (Figures S3–S10). Irradiation with visible light largely restores the initial spectrum, which parallels our electronic absorption spectroscopy results (*E*,*E*‐**oAzoBox** ^4+^: 64 %, *E*,*Z*‐**oAzoBox** ^4+^: 28 %, and and *Z*,*Z*‐**oAzoBox** ^4+^: 9 %). Smooth cycling between the two *E*‐ and *Z*‐predominant states was demonstrated without any noticeable degradation (Figure S11).

**Figure 3:**
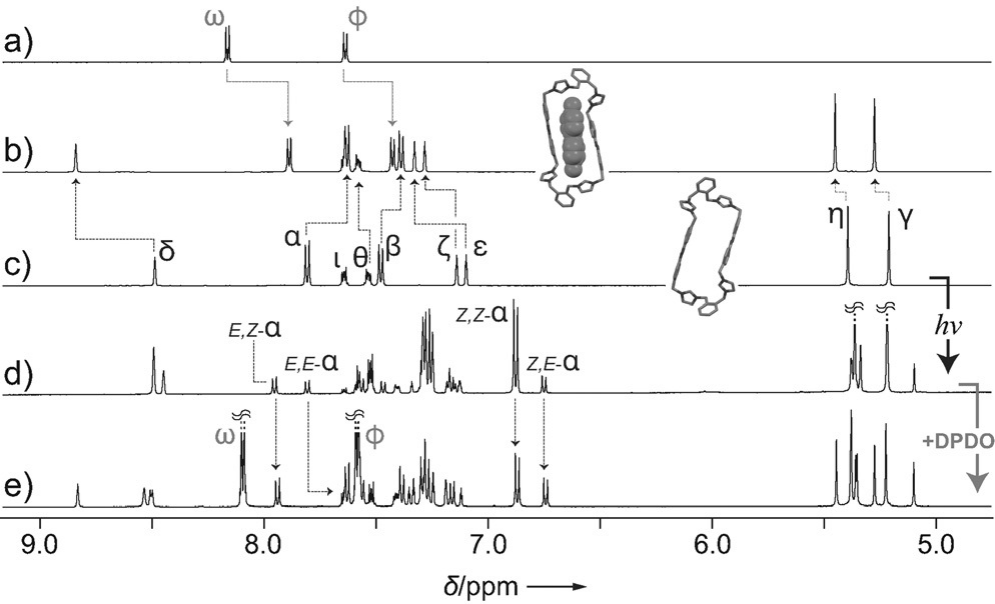
^1^H NMR spectra (CD_3_CN, 500 MHz) of **4DPDO** (a), *E*,*E*‐**oAzoBox**⊂**4DPDO** (b), *E*,*E*‐**oAzoBox** ^4+^ before (c) and after (d) UV light irradiation and followed by addition of excess **4DPDO** (e) (Hα resonances are assigned to illustrate binding behavior; for full assignments see the Supporting Information).

Thermal relaxation of the *Z*‐predominant **oAzoBox** ^4+^, via the stepwise pathway outlined in Figure S15, was monitored by thermal array ^1^H NMR spectroscopy at 313, 318, 323, and 328 K, as their stabilities are important considerations with regard to the potential of **oAzoBox** ^4+^ to act as a photoswitchable molecular container. Fitting the data to the appropriate kinetic models [Eqs. (S4)–(S7)] and the Eyring equation [Eqs. (S8), (S9)] allowed extraction of the rate constants *κ*_1_ and *κ*_2_ and the thermodynamic parameters Δ*G* ^≠^, Δ*H* ^≠^, and Δ*S* ^≠^ (Table 1, Figures S16–S34, Tables S1–S8).

**Table 1:** Thermodynamic data for the thermal *Z*→*E* isomerization of **oAzoBox** ^4+^ and **AzoBI** ^2+^ at 293 K.

Switching species	Δ*G* ^≠^ [kcal mol^−1^]	Δ*H* ^≠^ [kcal mol^−1^]	Δ*S* ^≠^ [cal mol^−1^ K^−1^]
^*Z*,*Z*→*E*,*Z*^ **oAzoBox** ^4+^	19.23±1.06	22.46±0.31	11.01±0.97
^*E*,*Z*→*E*,*E*^ **oAzoBox** ^4+^	22.24±4.53	23.85±2.30	5.48±7.85
^*Z*→*E*^ **AzoBI** ^2+^	19.86±4.2	22.66±1.21	9.55±3.77

The activation energy barrier (Δ*G* ^≠^) of *Z*,*Z*‐**oAzoBox** ^4+^→*E*,*Z*‐**oAzoBox** ^4+^ is very similar to that of *Z*‐**AzoBI** ^2+^→*E*‐**AzoBI** ^2+^ at 293 K (Figures S24, S34). Δ*G* ^≠^ of *E*,*Z*‐**oAzoBox** ^4+^→*E*,*E*‐**oAzoBox** ^4+^ is slightly higher at 22.24 kcal mol^−1^ (Figure S25), likely on account of the length disparity of *E*‐ and *Z*‐azobenzene. The magnitudes of the Δ*G* ^≠^ values were corroborated by computational studies (Tables S9, S10).

DFT calculations (B3LYP‐D3(BJ)/TZVP, Figure S41) revealed that the lowest energy molecular configuration of *Z*,*Z*‐**oAzoBox** ^4+^ is 10.97 kcal mol^−1^ greater than that of *E*,*Z*‐**oAzoBox** ^4+^, which is in turn 14.43 kcal mol^−1^ greater than that of *E*,*E*‐**oAzoBox** ^4+^. Such a result, combined with the similarity of the Δ*G* ^≠^ values (Tables 1), indicates that ring strain^31^ does not play a significant role in affecting the thermal isomerization mechanism for **oAzoBox** ^4+^.

The large size of *E*,*E*‐**oAzoBox** ^4+^ led us to consider whether small organic guest compounds could be accommodated within the cavity of the macrocycle. The representative compounds, 2,2′‐dipyridyl *N*,*N′*‐dioxide (**2DPDO**), 4,4′‐dipyridyl *N*,*N′*‐dioxide (**4DPDO**), 4‐phenylpyridine *N*‐oxide (**PPO**), and biphenyl‐4,4′‐dicarboxylate (**BPDC**) were chosen to test the interaction of *E*,*E*‐**oAzoBox** ^4+^ with hydrogen bond acceptor aromatic structures (Figure 1 c). ^1^H NMR revealed a binding interaction between *E*,*E*‐**oAzoBox** ^4+^ with **4DPDO**, **PPO** and **BPDC** (Figures 3 b, S36, and S37). However, no interaction was detected between *E*,*E*‐**oAzoBox** ^4+^ with **2DPDO**, presumably on account of steric effects. All cases of binding exhibited upfield shift perturbations of the aromatic resonances, H*α* and H*β*, whereas proton resonances H*γ*‐*ι* shifted downfield. The encapsulated guest proton resonances shifted upfield on account of the shielding by the azobenzene moieties at the long sides of *E*,*E*‐**oAzoBox** ^4+^.

**4DPDO** was selected as a representative example to illustrate the encapsulation potential of *E*,*E*‐**oAzoBox** ^4+^ in acetonitrile. A ^1^H NMR titration of **4DPDO** into *E*,*E*‐**oAzoBox** ^4+^ provided an association constant on the order of 10^3^ m ^−1^ (Figure 4) corresponding to an association free energy of about −4 kcal mol^−1^, which is consistent with the computationally obtained value of −4.06 kcal mol^−1^ (see the Supporting Information). The formation of *E*,*E*‐**oAzoBox** ^4+^⊂**4DPDO** was also confirmed by mass spectrometry (Figures 4 a, S38). A control ^1^H NMR experiment, whereby **4DPDO** was mixed with an equimolar amount of *E*‐**AzoBI** ^2+^, showed no evidence of interaction, suggesting that macrocyclic preorganization is a requirement for strong binding in our system (Figure S13). Similar conclusions were obtained from an analogous experiment with **4DPDO** and *α*,*α*′‐bis[3‐(1‐methylimidazolium)]‐*o*‐xylene, a model subcomponent analog of the *o*‐xylene bridging unit of **oAzoBox** ^4+^ (Figure S14).

**Figure 4:**
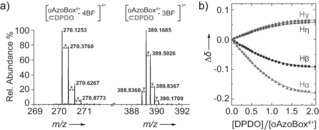
High‐resolution mass spectrometry of *E*,*E*‐**oAzoBox** ^4+^⊂**4DPDO** (average values: *x*=0.2507, *y*=0.3338) (a). Global fit by nonlinear regression of the ^1^H NMR shifts of the H*α*, H*β*, H*γ* and H*η* resonances to a 1:1 binding model^32^ (b).

The inclusion geometry assigned to the bimolecular complex was corroborated by quantum‐mechanical calculations (Figure 2). The energy‐minimized structure of *E*,*E*‐**oAzoBox** ^4+^⊂**4DPDO** shows that the container adopts a cagelike conformation with the phenyl rings of the azobenzene moieties lying in parallel planes and the **4DPDO** guest included in the cavity of the macrocycle. Each of the oxygen atoms of the guest are hydrogen‐bonded to two of the four of acidic H*δ* protons in an approximately symmetric fashion (Figure S42). This interpretation of the binding is supported by the significant downfield shift of the H*δ* proton resonance and a series of ^1^H NMR titration experiments (see the Supporting Information). Calculations also reveal that the macrocycle adopts a significantly expanded conformation in comparison to that of the solid‐state structure upon guest sequestration with an appreciably reduced aspect ratio of about 3. Similar conclusions were also established by quantum‐mechanical calculations for *E*,*E*‐**oAzoBox** ^4+^⊂**BPDC** (Figure S46).

Exposure of *E*,*E*‐**oAzoBox** ^4+^⊂**4DPDO** to UV light induces the *E*→*Z* isomerization of the host and the release of **4DPDO**, evidenced by the downfield shift of the H*ω* and H*ϕ* resonances (Figures 5 and S51). This result can be rationalized by assuming the **4DPDO** affinity of *E*,*Z*‐**oAzoBox** ^4+^ and *Z*,*Z*‐**oAzoBox** ^4+^ is negligible in comparison to that of *E*,*E*‐**oAzoBox** ^4+^. Indeed, when excess **4DPDO** was added into a *Z*‐predominant **oAzoBox** ^4+^ isomeric mixture, no evidence of interaction was detected between *Z*,*Z*‐**oAzoBox** ^4+^ and the guest molecule (unperturbed *Z*,*Z*‐H*α* resonances) and only extremely limited interaction was observed for *E*,*Z*‐ **oAzoBox** ^4+^ (Figures 3 d,e). An attempt to quantify the **4DPDO** affinity of the *E*,*Z* stereoisomers was unsuccessful on account of the limited interaction between the guest and the host after UV light irradiation. In any event, irradiating the mixture with visible light reverts the system back to the *E*,*E*‐**oAzoBox** ^4+^⊂**4DPDO** enriched state.

**Figure 5:**
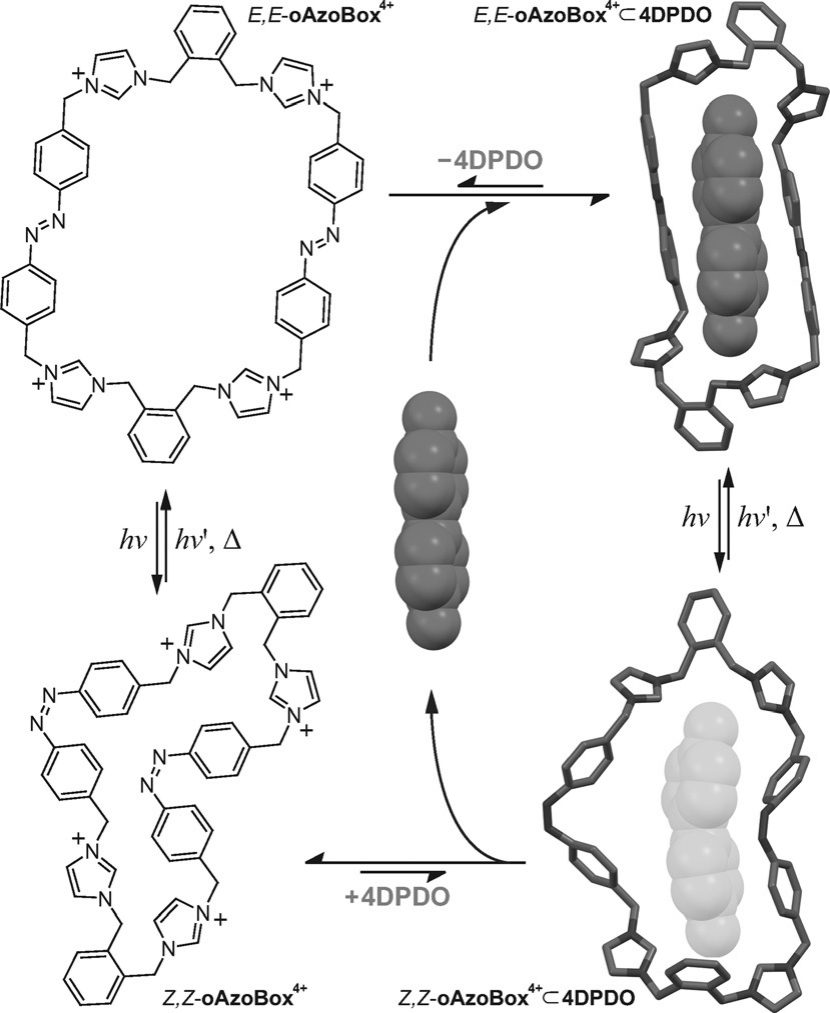
Schematic representation of the photocontrolled catch‐and‐release of **4DPDO** by **oAzoBox** ^4+^, inlcuding the geometry optimized molecular structures (B3LYP‐D3(BJ)/TZVP) of *E*,*E*‐**oAzoBox** ^4+^⊂**4DPDO** (top) and putative *Z*,*Z*‐**oAzoBox** ^4+^⊂**4DPDO** (bottom).

The thermal stability of *Z*‐predominant **oAzoBox** ^4+^ was unaffected by the presence of **4DPDO** (Figure S35). The low **4DPDO** affinity of *E*,*Z*‐**oAzoBox** ^4+^ and *Z*,*Z*‐**oAzoBox** ^4+^ can be attributed to a significant decrease in the size of the cavity of the macrocycle. Additionally, favorable orientation of the acidic H*δ* protons towards the interior of the cavity is lost upon UV light irradiation, and consequently the possibility of establishing concerted hydrogen‐bonding interactions between the host and guest. This was supported by calculated structures of *E*,*Z*‐**oAzoBox** ^4+^ and *Z*,*Z*‐**oAzoBox** ^4+^ (Figures S39, S40) and the high energy levels associated with the putative *E*,*Z*‐**oAzoBox** ^4+^⊂**4DPDO** and *Z*,*Z*‐**oAzoBox** ^4+^⊂**4DPDO** complexes (Figures S43–S45). The concept of photocontrolled catch‐and‐release was also demonstrated for **BPDC** (Figure S37).

In conclusion, we have demonstrated how the photochromic macrocycle, **oAzoBox** ^4+^, may be synthesized in three simple steps and allows for the realization of the remote controlled catch‐and‐release concept mediated by a photoswitchable molecular container. Our work illustrates how the incorporation of photochromic switching elements into a flexible macrocyclic framework, which does not compromise light‐induced isomerization, can also exhibit relevant recognition properties. These have been achieved by exploiting the hereto unreported complementary hydrogen‐bonding H‐donor imidizolium and H‐acceptor *N*‐oxide pairs, as well as the H‐acceptor carboxylate. The incorporation of molecular switches into optimized organic container structures may be regarded as a general approach to regulate encapsulation, in a non‐invasive fashion, of selected molecular species.

Such a strategy may also be used as a supramolecular host–guest logic system, as distinct switching between stereoisomeric mixtures may be achieved, where the predominant components posses association constants separated by orders of magnitude.

## Supporting information

As a service to our authors and readers, this journal provides supporting information supplied by the authors. Such materials are peer reviewed and may be re‐organized for online delivery, but are not copy‐edited or typeset. Technical support issues arising from supporting information (other than missing files) should be addressed to the authors.

Supplementary
