# Supplementary material for: A Dynamic and Responsive Host in Action: Light‐Controlled Molecular Encapsulation
Source: Angew Chem Int Ed Engl. 2016 Oct 28;55(52):16096–100. doi: 10.1002/anie.201607693 (PMC5396292; doi:10.1002/anie.201607693)
Supplement: Supplementary file 1 — Supplementary [file ANIE-55-16096-s001.pdf]

Angewandte Chemie
Eine Zeitschrift der Gesellschaft Deutscher Chemiker

Supporting Information

A Dynamic and Responsive Host in Action: Light-Controlled Molecular Encapsulation

Seán T. J. Ryan[+], Jesús del Barrio[+], Reynier Suardíaz, Daniel F. Ryan, Edina Rosta, and Oren A. Scherman**

anie_201607693_sm_miscellaneous_information.pdf

Contents

1 Materials and Methods **S4**

1.1 Materials S4

1.2 Nuclear Magnetic Resonance (NMR) Spectroscopy S4

1.3 Electronic Absorption (UV-vis) Spectroscopy S4

1.4 Computational Calculations S4

1.5 Kinetic Data Fitting for $Z \rightarrow E$ Thermal Isomerisation S4

2 Synthesis **S6**

2.1 Synthesis of 4,4'-[Bis(hydroxy)methyl]azobenzene (**1**) S6

2.2 4,4'-[Bis(imidazol-1-ylmethyl]azobenzene (**2**) S6

2.3 1,1'-[1,2-phenylenebis(methylene)]bis[3-methyl-imidazolium dihexafluorophosphate (**3**) S7

2.4 Synthesis of **oAzoBox**$^{4+}$$\cdot$**4BF**$_4$$^-$ S7

2.5 Synthesis of **AzoBI**$^{2+}$$\cdot$**2PF**$_6$$^-$ S8

3 Electronic Absorption Spectroscopy **S9**

3.1 Photoisomerisation of **AzoBI**$^{2+}$ S9

3.2 Photoisomerisation of **oAzoBox**$^{4+}$ S10

4 Nuclear Magnetic Resonance Spectroscopy **S11**

4.1 Two-Dimensional NMR S11

4.1.1 COSY ^{1}H NMR S11

4.1.2 ROESY ^{1}H NMR S14

4.2 One-Dimensional NMR S16

5 Kinetics and Thermodynamics of $E \rightarrow Z$ Thermal Isomerisation **S21**

5.1 Differential Equations for the thermal $Z \rightarrow E$ isomerisation of **oAzoBox**$^{4+}$. S21

5.1.1 oAzoBox^{4+}$\cdot$4BF$_4$$^-$ S22

5.1.2 AzoBI^{2+}$\cdot$2PF$_6$$^-$ S27

5.2 Additional Guests for **oAzoBox**$^{4+}$ S36

6 Electrospray Ionisation Mass Spectrometry **S36**

7 X-ray Crystallography **S37**

7.1 **oAzoBox**$^{4+}$$\cdot$**4BF**$_4$$^-$ S37

7.1.1 Crystallization Methods S37

7.1.2 X-ray Crystallography S37

7.1.3 Crystallographic Data S37

8 Computational Studies **S38**

8.1 Energy Minimised Structures of **oAzoBox**$^{4+}$ S38

8.2 Relaxed Potention Energy Surface Scans for Thermal $Z \rightarrow E$ Isomerisation of **oAzoBox**$^{4+}$ S41

8.3 Energy Minimised Structures of **oAzoBI**$^{2+}$ S42

9 Phototriggered Guest Release **S43**

1 Materials and Methods

1.1 Materials

All materials and anhydrous solvents were purchased from Aldrich and used as received. **1** was synthesised by a previously reported literature procedure[1]. **BPDC** was produced by dissolving biphenyl-4,4'-dicarboxylic acid in MeCN followed buy the addition of five equivalents of triethylamine, in line with previously reported procedures[2]

1.2 Nuclear Magnetic Resonance (NMR) Spectroscopy

^{1}H NMR spectra were recorded on a Bruker Avance 500 TCI Cryoprobe Spectrometer. Chemical Shifts are recorded in ppm (δ) in CD_3CN (internal reference set to δ 1.94 ppm). ^{13}C NMR (126 MHz) spectra were recorded using a Bruker Avance III QNP Cryoprobe with simultaneous decoupling of ^{1}H nuclei and externally referenced to TMS set to 0 ppm. Titrations were performed by using stock solutions of host and guest to make up samples of desired concentrations. All spectra were recorded at 298 K unless otherwise stated.

1.3 Electronic Absorption (UV-vis) Spectroscopy

Electronic absorption spectroscopy was performed on a Varian Cary 4000 UV-vis spectrophotometer at 298 K.

1.4 Computational Calculations

Gas phase geometry optimisations of the complex, host and guest molecules were performed using B3LYP functional in combination with TZVP and including the Grimme's D3 dispersion correction with Becke-Johnson damping[3]. Frequency calculations were performed at the same level of theory to obtain the thermostatistical corrections from energy to free energy in the rigid rotor/harmonic-oscillator approximation and including zero-point-vibrational energy in the gas phase at 298K and 1 atm ($G^T{}_{RRHO}$). Solvation free energy was obtained at the same level of theory and using acetonitrile SMD continuum model (Gacet). Association free energy ΔG_a is then calculated as the sum of those contributions to the gas phase association energy ΔE[4].

$$\Delta G_a = \Delta E + \Delta G^T{}_{RRHO} + \Delta G_{acet} \tag{S1}$$

The Δ symbol represents that the supramolecular approach ΔX=X(complex)-X(host)-X(guest) have been used. For *E,E*-**oAzoBox**$^{4+}$$\subset$**4DPDO** the calculated Gibbs energy of association is -4.06 kcal mol^{-1}, which is in accordance with our experimental association constant.

1.5 Kinetic Data Fitting for $Z \rightarrow E$ Thermal Isomerisation

The evolution of *E,Z*-**oAzoBox**$^{4+}$ at a given temperature was determined from the integrated intensities of its corresponding Hα resonances in the ^{1}H NMR spectra. However, as the decay of these states depends on the decay of *E,E*-**oAzoBox**$^{4+}$, the first step was to characterise its decay. This was done by fitting the integrated intensities of its Hα resonance as a function of time with the following equation S2.

$$A(t) = A_0 e^{-2k_1 t} + C \tag{S2}$$

A_0 is the initial concentration of *E,E*-**oAzoBox**$^{4+}$, k_1 is its decay rate, t is time, and C is a constant representing the background noise of our detector.

The model fitting throughout this work was conducted using the Levenberg-Marquardt algorithm (LMA). LMA is a least squares fitting algorithm - whereby the squares of the residuals between the data and the model are minimised - and is more robust than some other least squares fitting algorithms, e.g. the Gauss-Newton algorithm. However, LMA still only finds a local minimum in the solution space and so requires a sufficiently good guesses for the model parameters as initial input. We therefore visually compared each fit to the data and found them all to approximate the data well. The uncertainties of the derived fit parameters quoted in this study represent one standard deviation.

Having determined the initial concentration and decay rate of *E,E*-**oAzoBox**$^{4+}$, the evolution of *E,Z*-**oAzoBox**$^{4+}$ was then fit the following equation S3.

$$B^{ez}(t) = (B_0^{ez} + K_1 A_0)e^{-k_2^{ez} t} - K_1 A_0 e^{-2k_1 t} + C \tag{S3}$$

B_0^{ez} is the initial concentration of *E,Z*-**oAzoBox**$^{4+}$ (where the *E*-Hα resonance was monitored), k_2^{ez} is the decay rate of *E,Z*-**oAzoBox**$^{4+}$, $K_1 = k_1/(2k_1 - k_2^{ez})$, and other variables have the same meaning as in Equation S2. When fitting, A_0 and k_1 were fixed as the best fit values obtained when fitting the evolution of *E,E*-**oAzoBox**$^{4+}$ with Equation S2. However, C was allowed to vary to account for any change in the noise of our detector between the *E,E*-**oAzoBox**$^{4+}$ and *E,Z*-**oAzoBox**$^{4+}$ measurements. Equation S3 was then fit for *Z,E*-**oAzoBox**$^{4+}$ (where the *Z*-Hα resonance was monitored), which hence allowed us to obtain values for B_0^{ze} and k_2^{ze}. Finally the combined evolution of *E,Z*-**oAzoBox**$^{4+}$ and *Z,E*-**oAzoBox**$^{4+}$ resonances was found by taking the average of the k_2^{ez} and k_2^{ze}, which we defined as k_2.

This process described was repeated for the *E,E*, *E,Z*, and *Z,E* isomers at different temperatures.

2 Synthesis

2.1 Synthesis of 4,4'-[Bis(hydroxy)methyl]azobenzene (1)

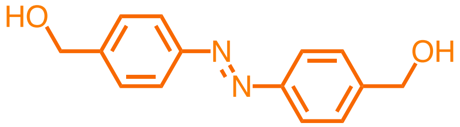

4-nitrobenzyl alcohol (10.0 g, 65.3 mmol), NaOH (4.0 g, 100.0 mmol) and Zn (5.0g, 76.5 mmol) in CH_3OH (40.0 mL) and H_2O (10,0 mL) were heated at reflux for 2 h. The reaction liquor was then filtered while hot and the precipitate was washed with warm CH_3OH (20 mL). The CH_3OH was then distilled from the filtrate and the residual suspension was neutralised with 1M HCl. The resulting orange precipitate was collected by filtration and washed with copious amount of H_2O. The residual solid was recrystallised from methanol (3.0 g, 38%).

^{1}H NMR (500 MHz, $(CD_3)_2SO$, *E*-isomer) δ (ppm) = 7.86 (d, 4H, J = 8.5 Hz), 7.53 (d, 4H, J = 8.5 Hz), 5.37 (t, 2H J = 5.8 Hz), 4.60 (d, 4H, J = 5.8 Hz);
^{13}C NMR (126 MHz, $(CD_3)_2SO$, *E*-isomer) δ (ppm) = 150.85, 146.26, 127.12, 122.35, 62.45.

2.2 4,4'-[Bis(imidazol-1-ylmethyl]azobenzene (2)

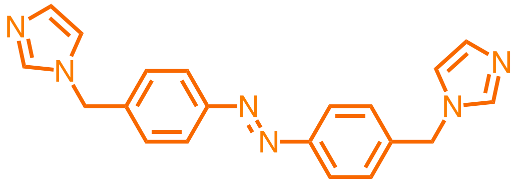

1 (1.0 g, 4.1 mmol) and 1,1'-carbonyldiimidazole (1.7 g, 10.5 mmol) in 1-methyl-2-pyrrolidinone (20.0 mL) was heated at 170 °C for 1 h. After cooling to RT, the reaction mixture was diluted with ethyl acetate (60.0 mL) and washed with H_2O (2 x 40.0 mL), brine (40.0 mL) and dried over $MgSO_4$. The organic phase was filtered and the solvent was distilled from the filtrate. The residual solid was recrystallised from ethanol (1.0 g, 71%).

^{1}H NMR (500 MHz, $(CD_3)_2SO$, *E*-isomer) δ (ppm) = 7.87 (d, 4H, J = 8.5 Hz), 7.57 (m, 2H), 7.26 (d, 4H, J = 8.5 Hz), 7.10 (m, 2H), 6.91 (m, 2H), 5.18 (s, 4H);
^{13}C NMR (126 MHz, $(CD_3)_2SO$, *E*-isomer) δ (ppm) = 150.61, 143.92, 141.54, 130.23, 125.97, 122.81, 122.12, 52.35;

2.3 1,1'-[1,2-phenylenebis(methylene)]bis[3-methyl-imidazolium dihexafluorophosphate (3)

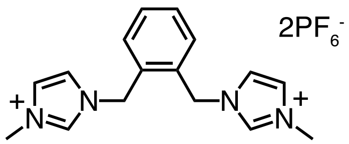

α,α'-Dibromo-*o*-xylene (1.5 g, 5.68 mmol) and N-methylimidiazole (1.87 g, 22.7 mmol) in CH_3CN (60 mL) were heated at reflux for 24 h. Upon cooling to room temperature, the white precipitate was filtered, washed with CH_2Cl (30 mL) and dried in air. The solid was then dissolved in water to which a saturated aqueous solution of ammonium hexafluorophosphate was added. The resulting precipitate was filtered and washed with H2O (50 mL) and CH_3OH (50 mL) and dried in air (2.32 g, 3.97 mmol, 70%).

^1H NMR (500 MHz, CD_3CN) δ (ppm) = 8.32 (s, 2H), 7.56-7.51 (m, 2H), 7.38 (m, 2H), 7.32-7.27 (b, 4H, two overlapping peaks), 5.35 (s, 4H), 3.82 (s, 6H);
^{13}C NMR (126 MHz, CD_3CN) δ (ppm) = 137.8, 133.3, 131.8, 131.8, 125.7, 124.0, 51.3, 37.6;
ESI-MS: m/z = 134.0838 [**3** - $2PF_6^-]^{2+}$, 413.1324 [**3** - $PF_6^-]^+$; found, 134.0842 [**3** - $2PF_6^-]^{2+}$, 413.1334 [**3** - $PF_6^-]^+$.

2.4 Synthesis of oAzoBox$^{4+}\cdot 4BF_4^-$

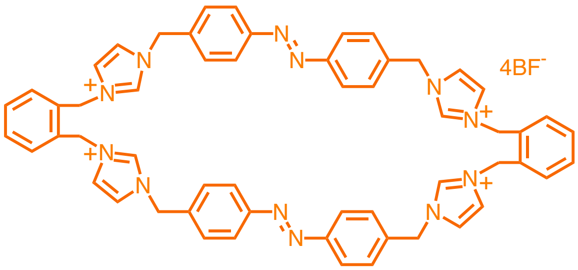

α,α'-dibromo-*o*-xylene (135 mg, 0.51 mmol) in CH_3CN (60 mL) was added dropwise to **2** (175 mg, 0.51 mmol) in CH_3CN (80 mL) over 6 h at RT with stirring in darkness. The solution was heated at reflux for 48 h. Upon cooling to RT, the solvent was removed under reduced pressure *via* rotary evaporation. H_2O (80 mL) was added to the residue and sonicated for 10 minutes to give an orange suspension, which was subjected to centrifugation. $AgBF_4$ (317 mg, 1.63 mmol) in H_2O (10 mL) was then added to the supernatant in the dark, resulting in the precipitation of AgBr. The suspension was subjected to centrifugation, after which the supernatant was set aside. The solid residue was then washed with H_2O (30 mL) and centrifuged to obtain the supernatant, which was combined with the previous supernatant. This procedure was repeated three times.

The combined supernatant was stirred at room temperature in the light for 48 h, after which the H_2O was removed *via* freeze drying. CH_3CN (70 mL) was then added to the residue, which was then centrifuged and the supernatant filtered through a 13 mm 0.45 μm

PTFE syringe filter. The filtrate was then heated to reflux for 10 minutes. Upon cooling to RT in the dark, the solvent was removed *via* rotary evaporation and the residue dried *in vacuo*. The crude product was then purified *via* recrystallisation (slow vapour diffusion of *i*-Pr_2O into CH_3CN, 10 mM) to yield **oAzoBox**$^{4+}$·**4BF**$_4^-$ (74 mg, 24%).

^{1}H NMR (500 MHz, CD_3CN) δ (ppm) = 8.48 (s, 4H), 7.81 (d, 8H, J = 8.4 Hz), 7.66-7.62 (m, 4H), 7.55-7.51 (m, 4H), 7.48 (d, 8H, J = 8.4 Hz), 7.14 (m, 4H), 7.10 (m, 4H), 5.39 (s, 8H), 5.21 (s, 8H);
^{13}C NMR (126 MHz, CD_3CN) δ (ppm) = 153.5, 137.4, 136.8, 133.1, 132.6, 131.9, 131.0, 124.4, 123.7, 123.4, 53.4, 51.4;
ESI-MS: m/z = 223.1104 [**oAzoBox**$^{4+}$·**4BF**$_4^-$ - $4BF_4^-$]$^{4+}$, 326.4817 [**oAzoBox**$^{4+}$·**4BF**$_4^-$ - $3BF_4^-$]$^{3+}$, 533.2243 [**oAzoBox**$^{4+}$·**4BF**$_4^-$ - $2BF_4^-$]$^{2+}$; found, 223.1101 [**oAzoBox**$^{4+}$·**4BF**$_4^-$ - $4BF_4^-$]$^{4+}$, 326.4812 [**oAzoBox**$^{4+}$·**4BF**$_4^-$ - $3BF_4^-$]$^{3+}$, 533.2240 [**oAzoBox**$^{4+}$·**4BF**$_4^-$ - $2BF_4^-$]$^{2+}$.

2.5 Synthesis of AzoBI^{2+}·2PF$_6^-$

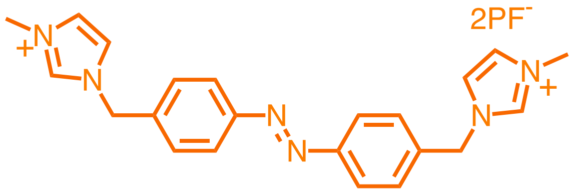

2 (40 mg, 0.12 mmol) and MeI (497 mg, 3.5 mmol) in CH_3CN (15 mL) were heated at reflux for 24 h. Upon cooling to RT the precipitate was filtered, washed with cold CH_2Cl_2 (3 x 15 mL) and dried *in vacuo*. The solid was then dissolve in H_2O (10 mL) to which a solution of saturated, aqueous ammonium hexfluorophosphate (0.5 mL) was added. The resulting precipitate was filtered, washed with H_2O (2 x 10 mL) and CH_3OH (1 x 5 mL) and dried *in vacuo* to yield **AzoBI**$^{2+}$·**2PF**$_6^-$ (66.5 mg, 86%).

^{1}H NMR (500 MHz, CD_3CN) δ (ppm) = 8.49 (s, 2H), 7.94 (d, 4H, J = 8.5 Hz), 7.56 (d, 4H, J = 8.5 Hz), 7.40 (m, 2H), 7.37 (m, 2H), 5.41 (s, 4H), 3.83 (s, 6H);
^{13}C NMR (126 MHz, CD_3CN) δ (ppm) = 153.7, 138.0, 137.3, 130.7, 125.2, 124.3, 123.4, 53.3, 37.0.
ESI-MS: m/z = 186.1026 [**AzoBI**$^{2+}$·**2PF**$_6^-$ - $2PF_6^-$]$^{2+}$, 517.1699 [**AzoBI**$^{2+}$·**2PF**$_6^-$ - PF_6^-]$^+$; found, 186.1021 [**AzoBI**$^{2+}$·**2PF**$_6^-$ - $2PF_6^-$]$^{2+}$, 517.1684[**AzoBI**$^{2+}$·**PF**$_6^-$ - PF_6^-]$^+$.

3 Electronic Absorption Spectroscopy

3.1 Photoisomerisation of AzoBI^{2+}

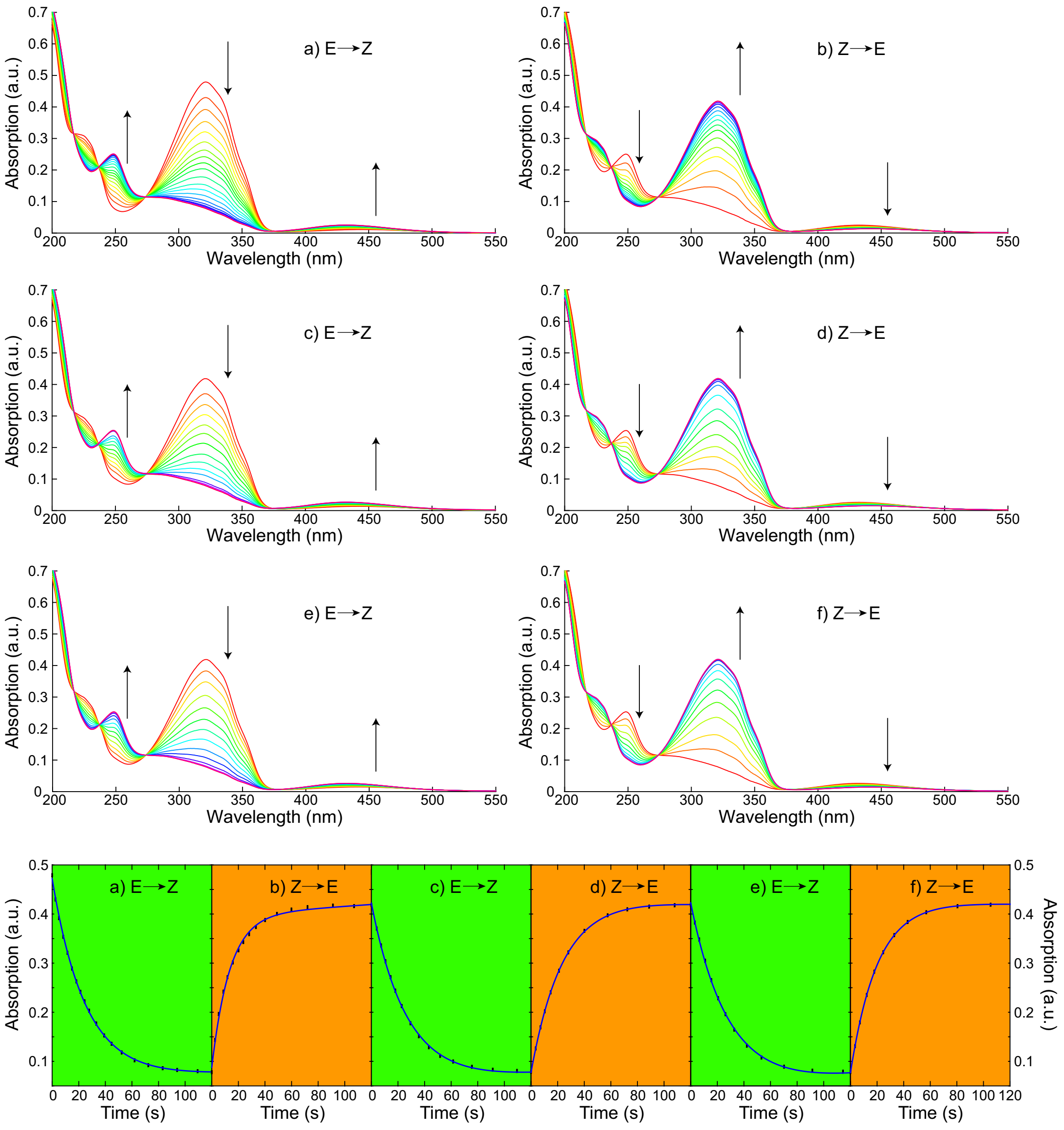

Figure S1. Electronic absorption spectra of **AzoBI**$^{2+}$ (CH_3CN) upon increased irradiation at 350 nm to achieve the Z-photostationary state (**a**, **c** and **e**) and at 420 nm to achieve the E-photostationary state (**b**, **d** and **f**) (top) and the kinetic profiles of the $E{\rightarrow}Z$ photoisomerisation as monitored by the change in optical density at 320 nm (bottom).

3.2 Photoisomerisation of oAzoBox^{4+}

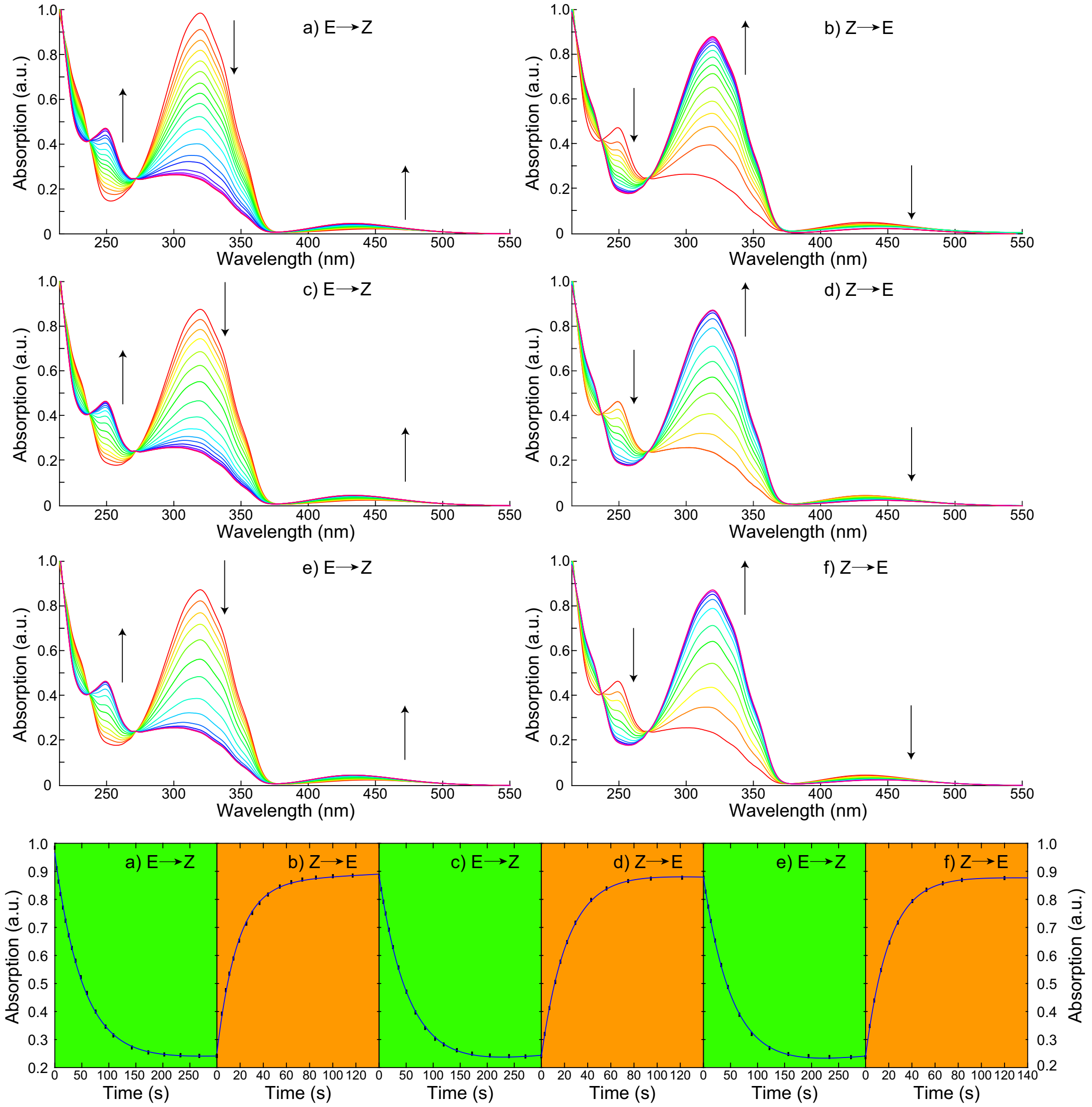

Figure S2. Electronic absorption spectra of **oAzoBox**$^{4+}$ (CH_3CN) upon increased irradiation at 350 nm to achieve the Z-photostationary state (**a**, **c** and **e**) and at 420 nm to achieve the E-photostationary state (**b**, **d** and **f**) (top) and the kinetic profiles of the $E \rightarrow Z$ photoisomerisation as monitored by the change in optical density at 320 nm (bottom).

4 Nuclear Magnetic Resonance Spectroscopy

4.1 Two-Dimensional NMR

4.1.1 COSY ^{1}H NMR

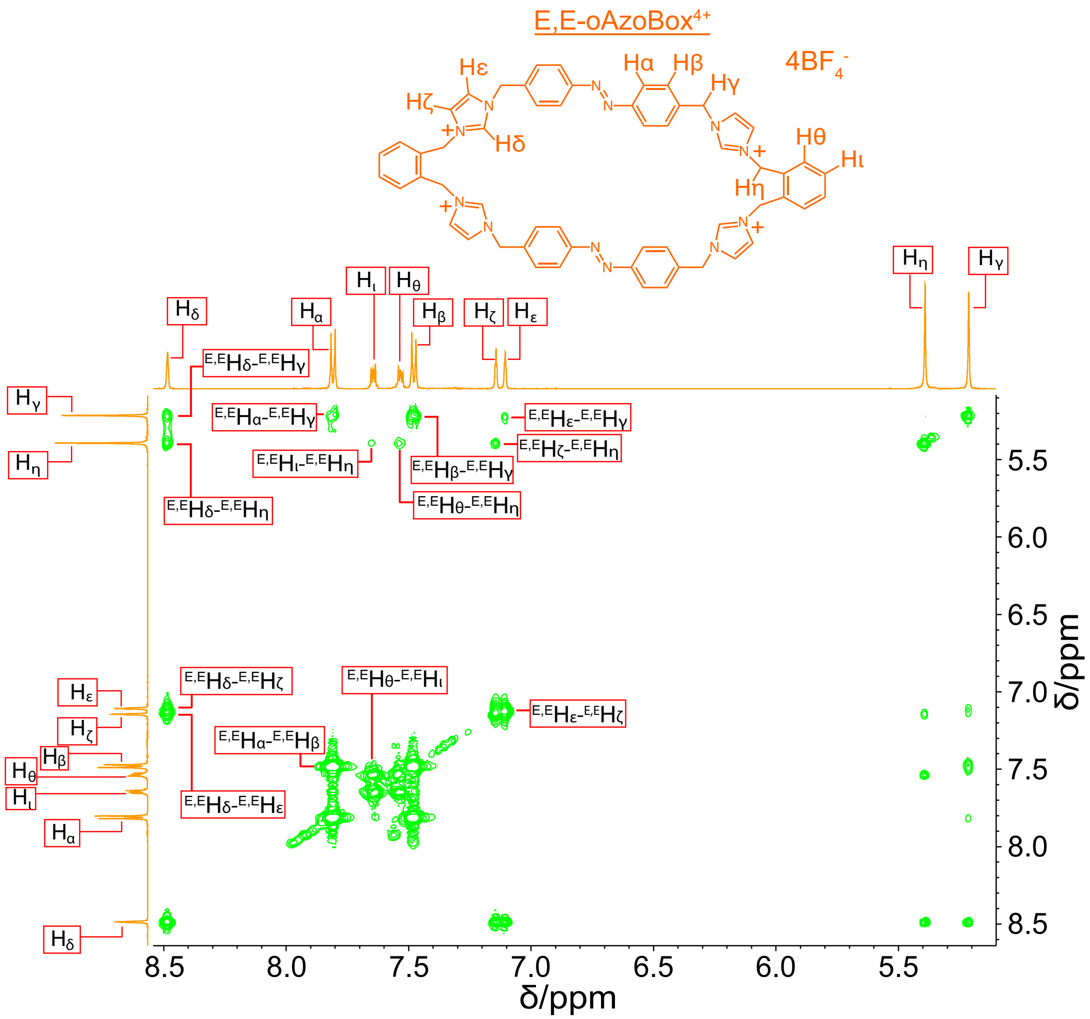

Figure S3. COSY ^{1}H NMR spectrum (CD_3CN, 500 MHz) of **oAzoBox**$^{4+}$.

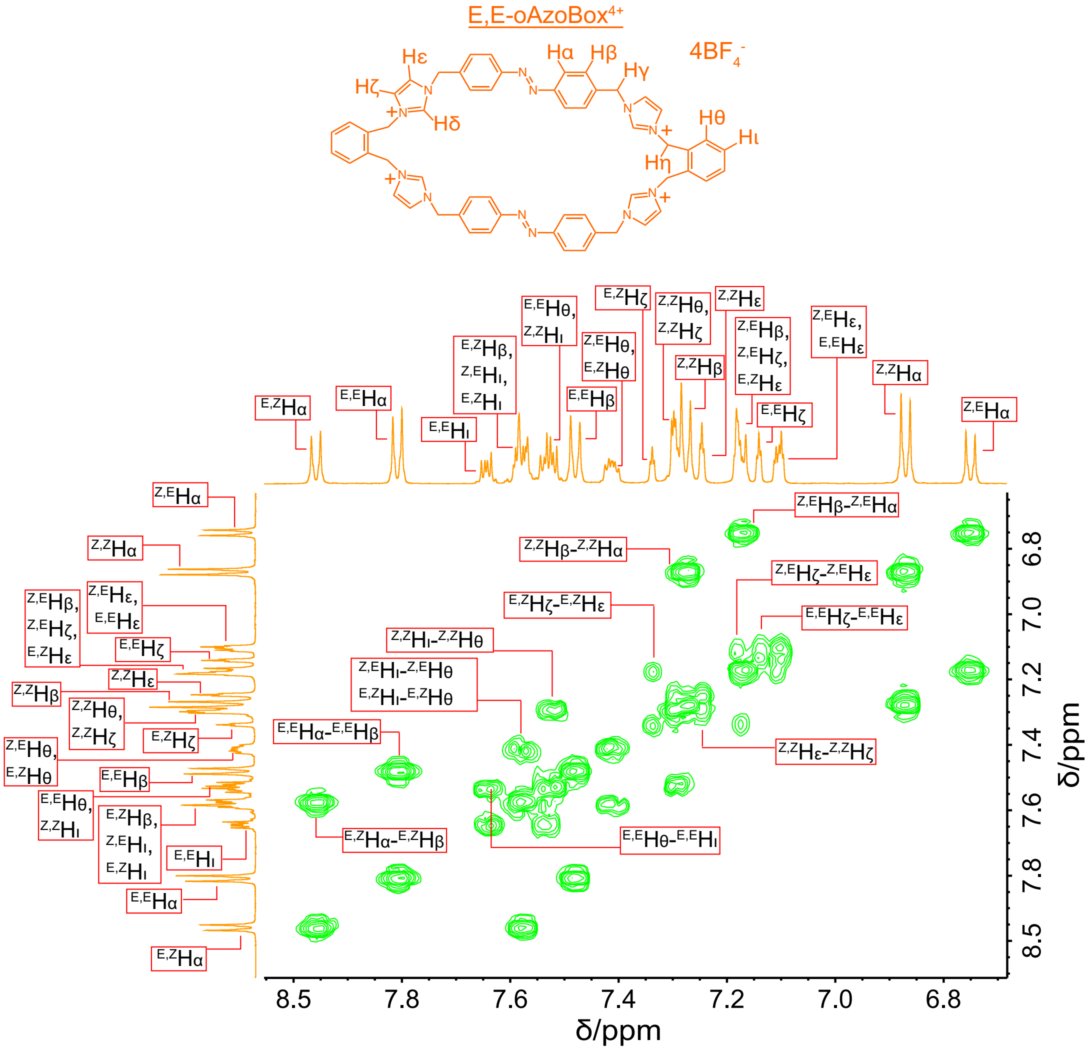

,

Figure S4. COSY ^{1}H NMR spectrum (CD_3CN, 500 MHz, aromatic-aromatic resonance correlation) of **oAzoBox**$^{4+}$ upon partial *Z*-conversion *via* irradiation at 350 nm.

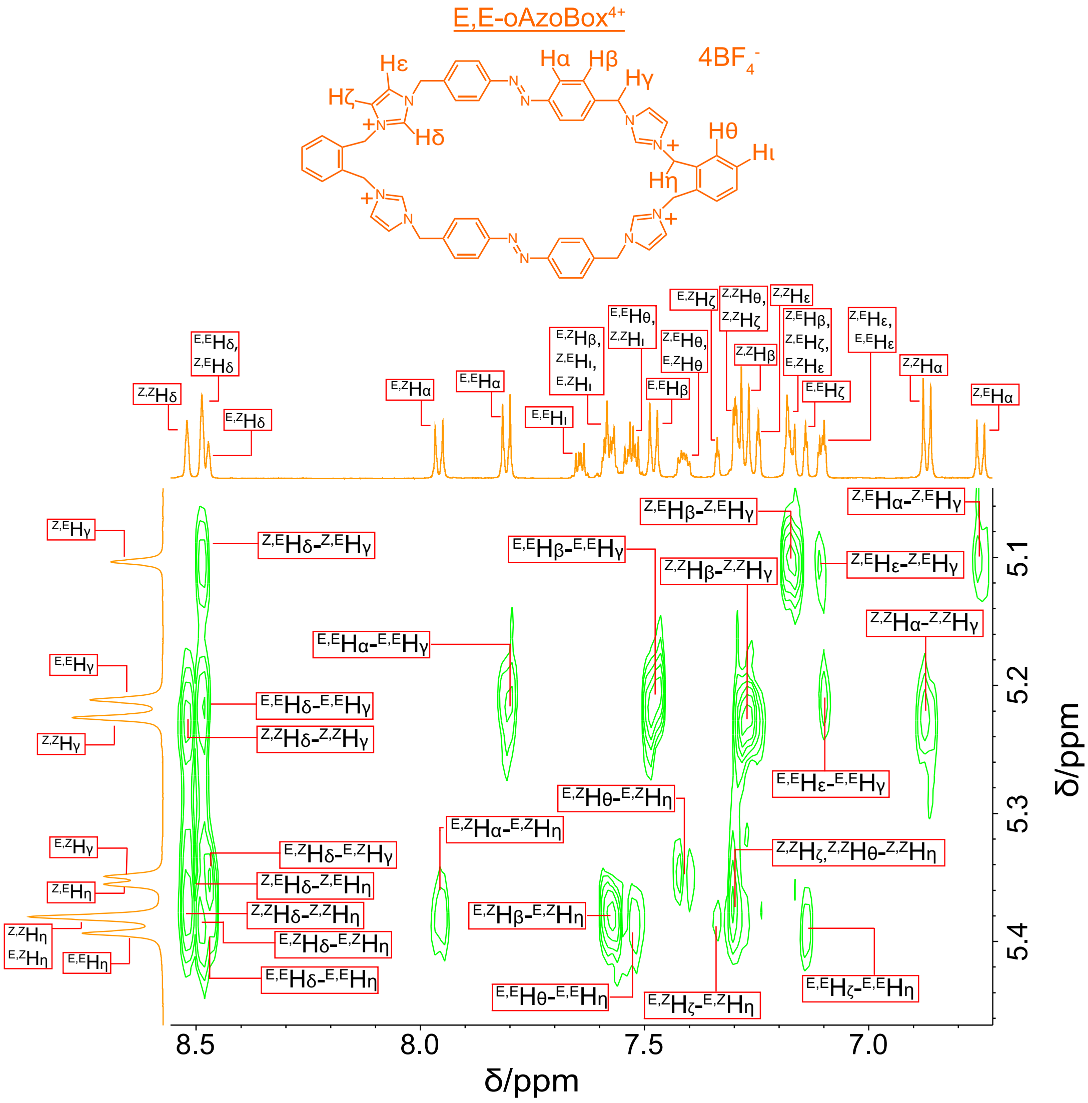

Figure S5. COSY ^{1}H NMR spectrum (CD_3CN, 500 MHz, aromatic-aliphatic resonance correlation) of **oAzoBox**$^{4+}$ upon partial *Z*-conversion *via* irradiation at 350 nm.

4.1.2 ROESY ^{1}H NMR

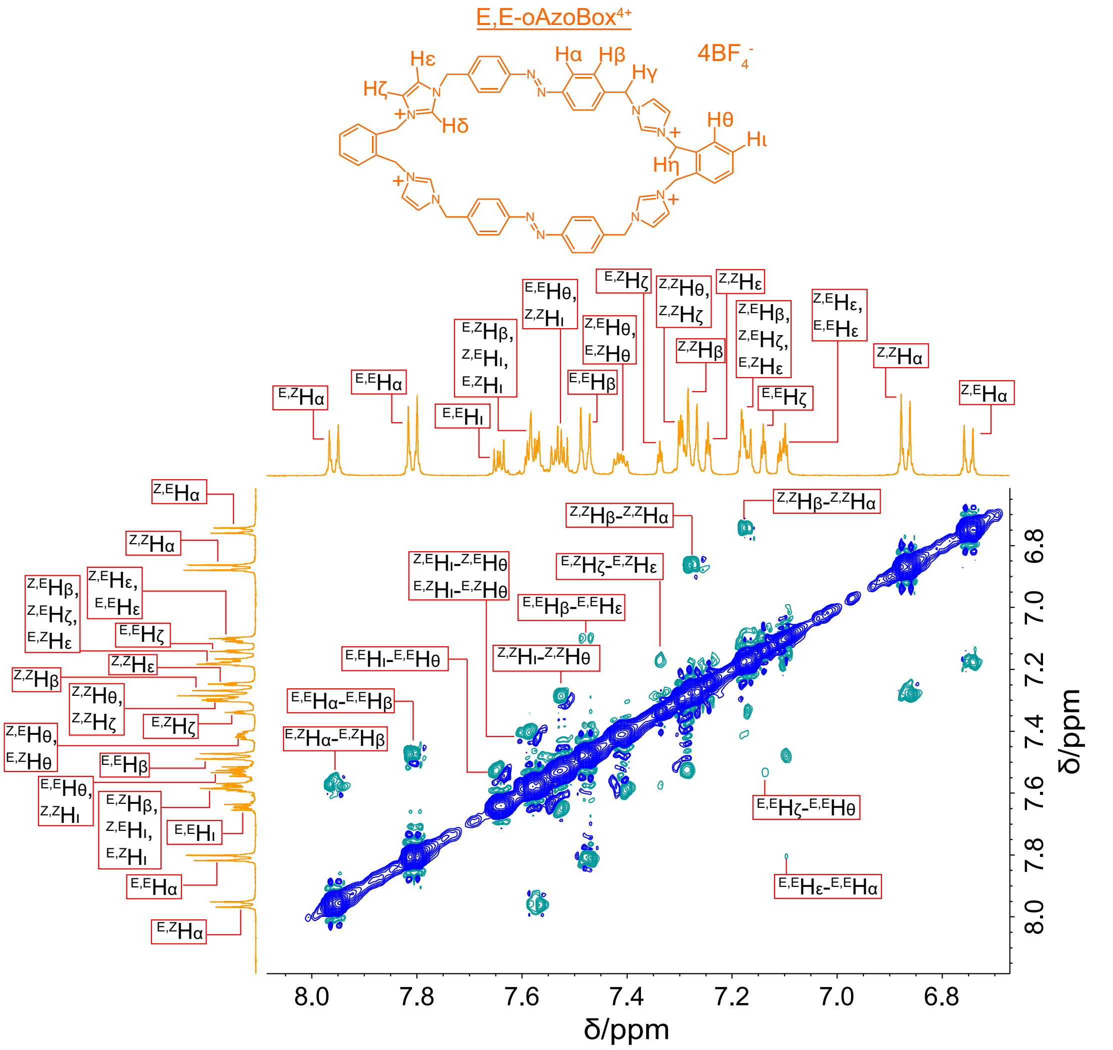

Figure S6. ROESY ^{1}H NMR spectrum (CD_3CN, 500 MHz, aromatic-aromatic resonance correlation) of **oAzoBox**$^{4+}$ upon partial Z-conversion *via* irradiation at 350 nm.

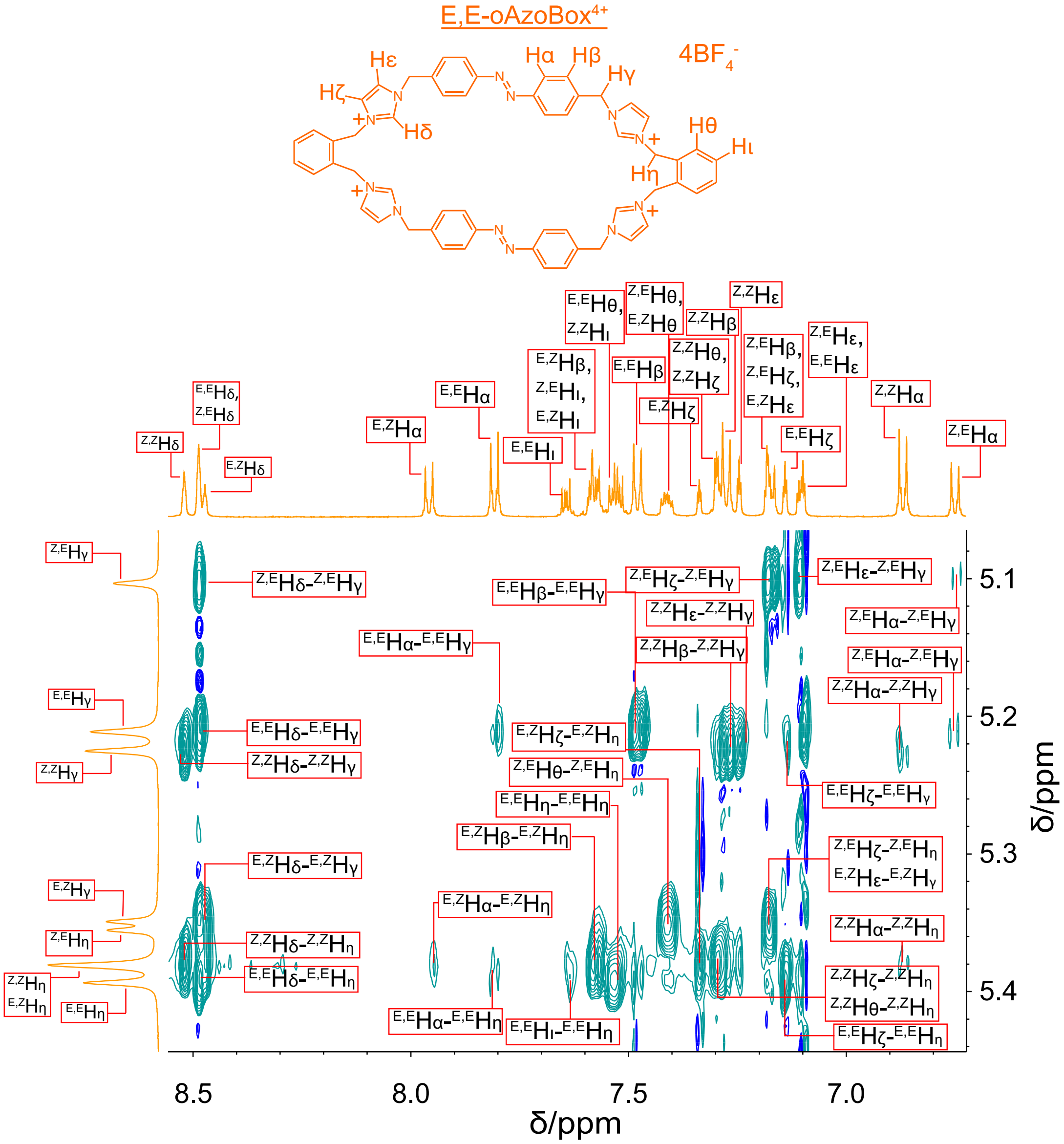

Figure S7. ROESY ^{1}H NMR spectrum (CD_3CN, 500 MHz, aromatic-aliphatic resonance correlation) of **oAzoBox**$^{4+}$ upon partial *Z*-conversion *via* irradiation at 350 nm.

4.2 One-Dimensional NMR

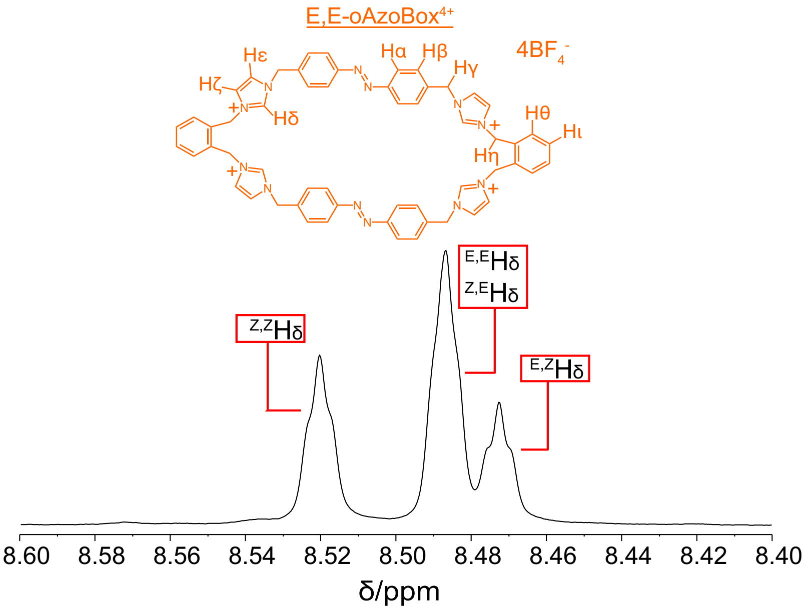

Figure S8. ^{1}H NMR spectrum (CD_3CN, 500 MHz, aromatic region between 8.4-8.6 ppm) with resonance assignments of **oAzoBox**$^{4+}$ upon partial Z-conversion *via* irradiation at 350 nm.

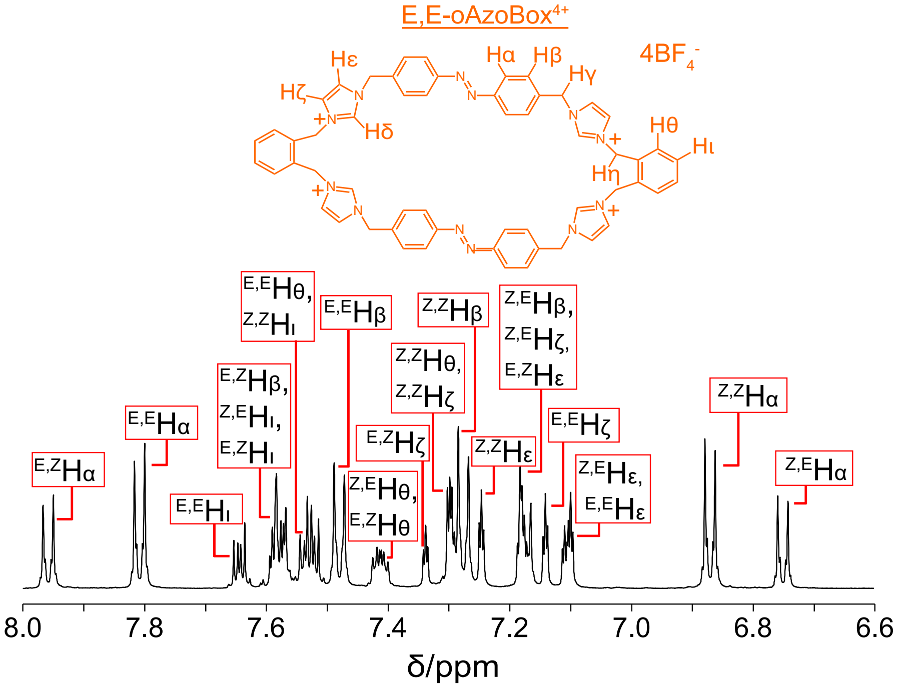

Figure S9. ^{1}H NMR spectrum (CD_3CN, 500 MHz, aromatic region between 6.6-8.0 ppm) with resonance assignments of **oAzoBox**$^{4+}$ upon partial Z-conversion *via* irradiation at 350 nm.

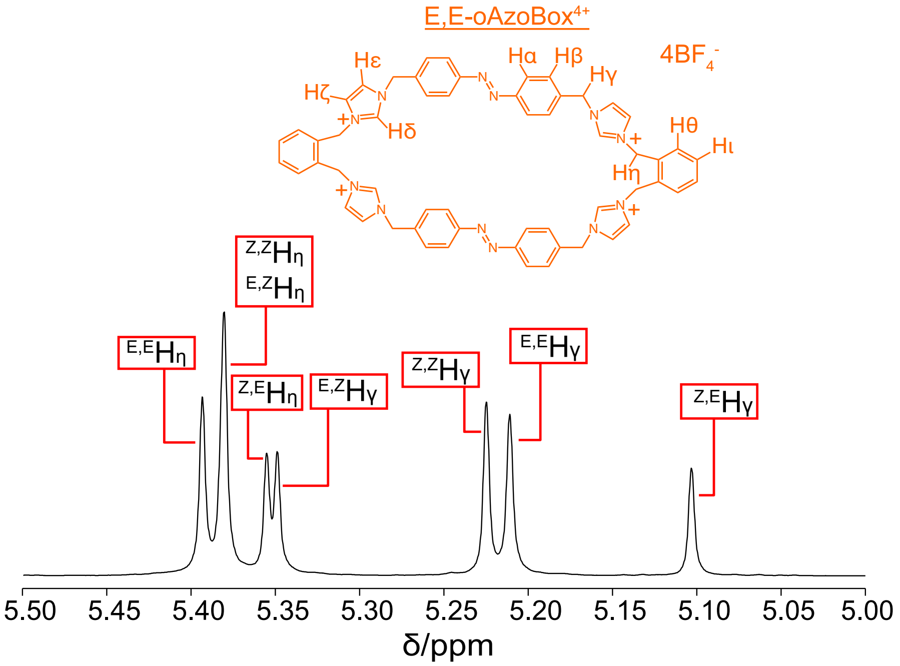

Figure S10. ^{1}H NMR spectrum (CD_3CN, 500 MHz, aliphatic region between 5.0-5.0 ppm) with resonance assignments of **oAzoBox**$^{4+}$ upon partial Z-conversion *via* irradiation at 350 nm.

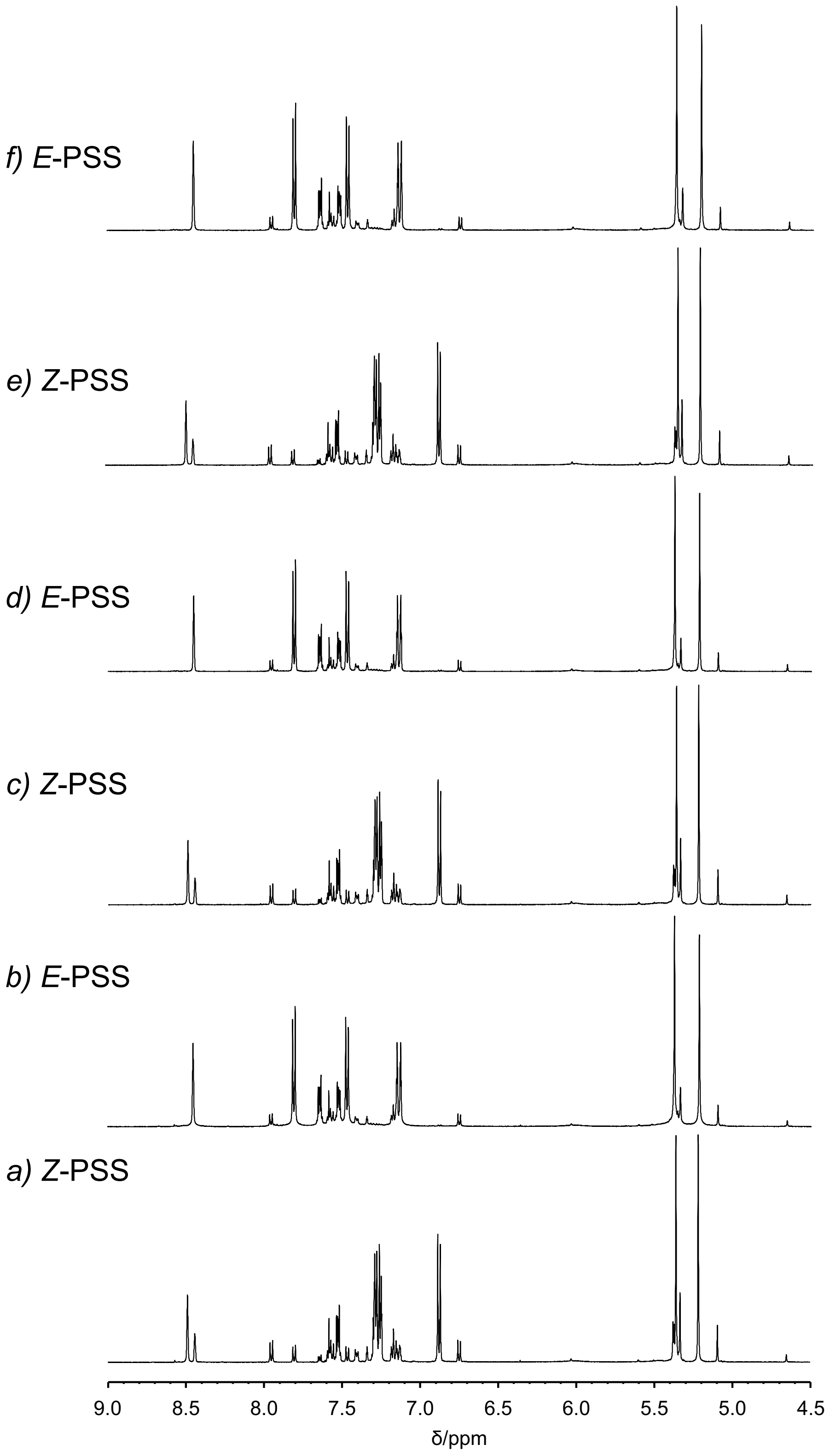

Figure S11. ^{1}H NMR spectra (CD_3CN, 500 MHz) of **oAzoBox**$^{4+}$ upon alternating irradiation at 350 nm to achieve the Z-**PSS** (**a**, **c** and **e**) and at 420 nm to achieve the E-**PSS** (**b**, **d** and **f**).

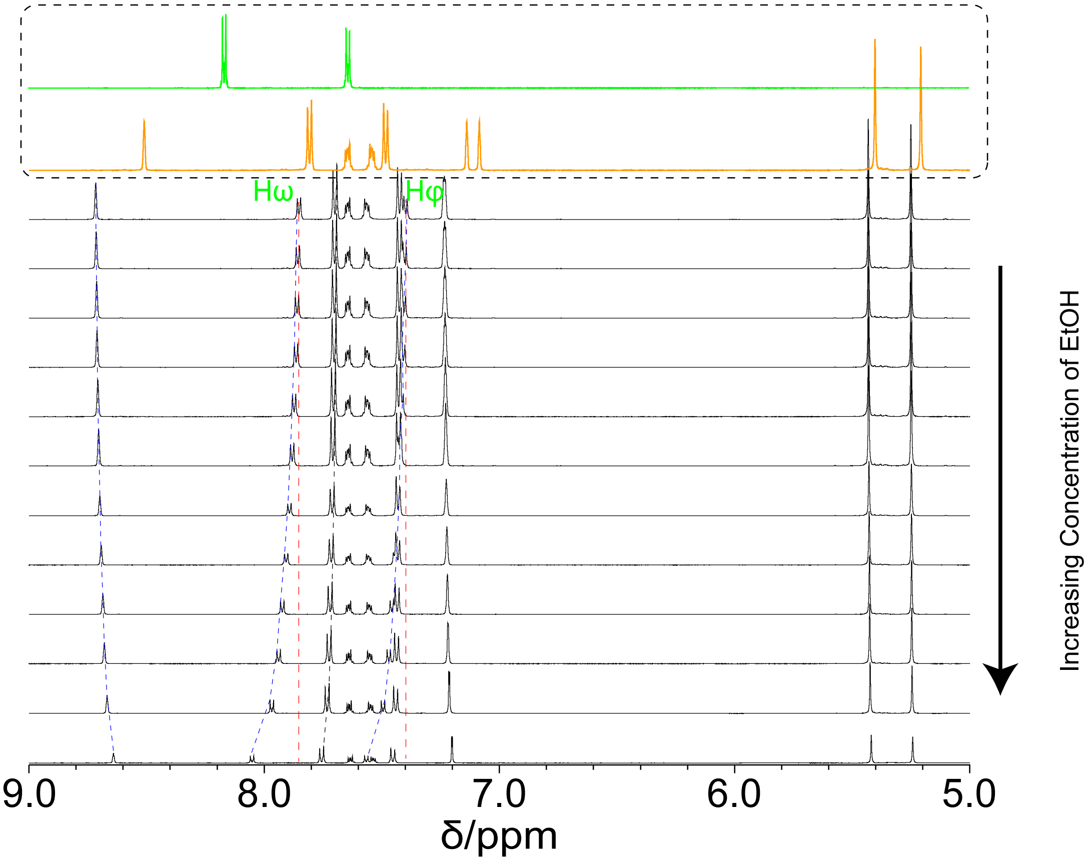

Figure S12. ^{1}H NMR titration (CD_3CN, 500 MHz) of EtOH into *E,E*-**oAzoBox**$^{4+}$⊂**4DPDO**. Blue dotted lines track the shifting proton resonances of **4DPDO** and *E,E*-H_δ and the red lines indicate the non-shifted **4DPDO** proton resonances. The green and orange spectra (CD_3CN, 500 MHz, top) are that of **4DPDO** and *E,E*-**oAzoBox**$^{4+}$, respectively.

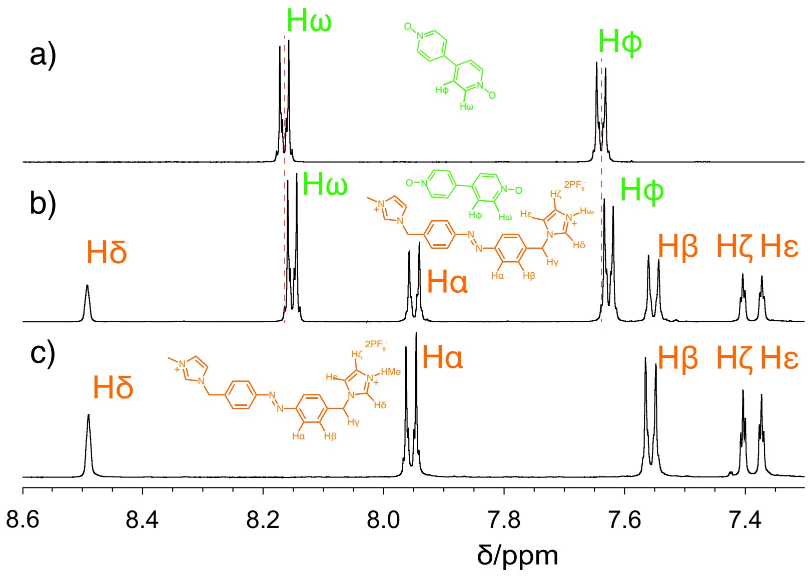

Figure S13. ^{1}H NMR (CD_3CN, 500 MHz) of (a) **4DPDO**, (b) 1:1 *E*-**AzoBI**$^{2+}$:**4DPDO** and (c) *E*-**AzoBI**$^{2+}$.

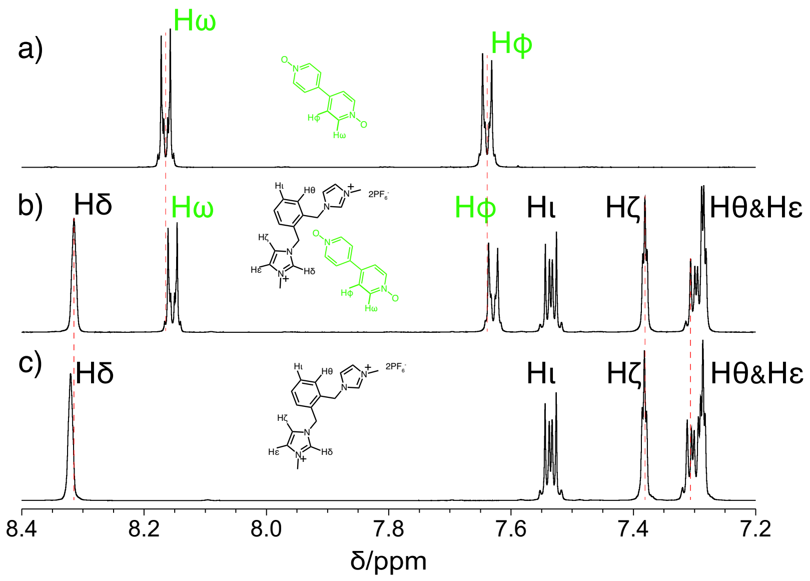

Figure S14. ^{1}H NMR (CD_3CN, 500 MHz) of (a) **4DPDO**, (b) 1:1 **3**:**4DPDO** and (c) **3**.

5 Kinetics and Thermodynamics of *E*→*Z* Thermal Isomerisation

$$\text{Z,Z-oAzoBox}^{4+} \xrightarrow{2\kappa_1} \text{E,Z-oAzoBox}^{4+} \xrightarrow{\kappa_2} \text{E,E-oAzoBox}^{4+}$$

Figure S15. Scheme illustrating the thermal $Z \rightarrow E$ isomerization of **oAzoBox**$^{4+}$. As either of two azobenzene units may undergo the initial $Z \rightarrow E$ isomerization in *Z,Z*-**oAzoBox**$^{4+}$, its the observed decay of is twice that of the actual rate of thermal isomerization of its two azobenzene components (κ_1). *E,Z*-**oAzoBox**$^{4+}$ may be equivalently written as *Z,E*-**oAzoBox**$^{4+}$.

5.1 Differential Equations for the thermal *Z*→*E* isomerisation of oAzoBox^{4+}

$$A(t) = A_0 e^{-2\kappa_1 t} + C_a \tag{S4}$$

$$B(t) = (B_0 + A_0 \frac{\kappa_1}{2\kappa_1 - \kappa_2}) e^{-\kappa_2 t} - \frac{\kappa_1}{2\kappa_1 - \kappa_2} A_0 e^{-2\kappa_1 t} + C_b \tag{S5}$$

$$\frac{dA(t)}{dt} = A'(t) = -2\kappa_1 A(t) \tag{S6}$$

$$\frac{dB(t)}{dt} = B'(t) = 2\kappa_1 A(t) - \kappa_2 B(t) \tag{S7}$$

$$\kappa = \frac{\kappa_B T}{h} e^{\frac{-\Delta H^\ddagger}{RT}} e^{\frac{-\Delta S^\ddagger}{R}} \tag{S8}$$

$$\ln \frac{\kappa h}{T \kappa_B} = \frac{-\Delta H^\ddagger}{R} \cdot \frac{1}{T} + \frac{-\Delta S^\ddagger}{R} \tag{S9}$$

A(t) represents the concentration of *Z,Z*-**oAzoBox**$^{4+}$, B(t) represents the concentration of *E,Z*-**oAzoBox**$^{4+}$ (which is equivalent to *Z,E*-**oAzoBox**$^{4+}$). C_a and C_b are constants.

5.1.1 oAzoBox^{4+}·4BF$_4^-$

313 K

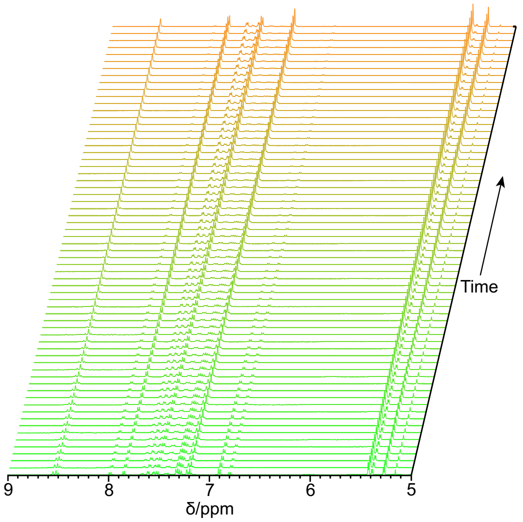

Figure S16. Temporal ^{1}H NMR thermal relaxation spectra (CD$_3$CN, 500 MHz, 313 K) of Z-predominant **oAzoBox**$^{4+}$, obtained by irradiation at 350 nm.

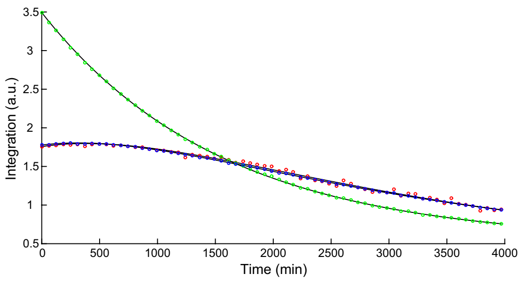

Figure S17. Kinetic fit of selected Hα proton resonances of the temporal ^{1}H NMR thermal relaxation spectra (CD$_3$CN, 500 MHz, 313 K) of Z-predominant **oAzoBox**$^{4+}$, obtained by irradiation at 350 nm. The integrations of the proton resonances were fitted to equations S6 and S7.

Table S1. Rate (κ, min^{-1}) and time constants (τ, min) for the rise and decay of the Hα proton resonances belonging to E,E-**oAzoBox**$^{4+}$, E,Z-**oAzoBox**$^{4+}$ and Z,Z-**oAzoBox**$^{4+}$ as measured by ^{1}H NMR (CD$_3$CN, 500 MHz, 313 K)

Proton	Shift [δ, ppm]	Rate Constant [κ, min^{-1}]	Error in κ [$\Delta\kappa$, min^{-1} x 10^{-6}]	Time Constant [τ, min]
E,Z-Hα	7.984	0.000539	16.6	1853.70
Z,Z-Hα	6.904	0.000639^a	0.948	1564.95
Z,E-Hα	6.798	0.000597	3.41	1674.48

^{a} Fit of the total integration area of the Z,Z-Hα proton resonance, such that the obtained rate constant is twice that of a single **AB** unit ($2\kappa_1$).

318 K

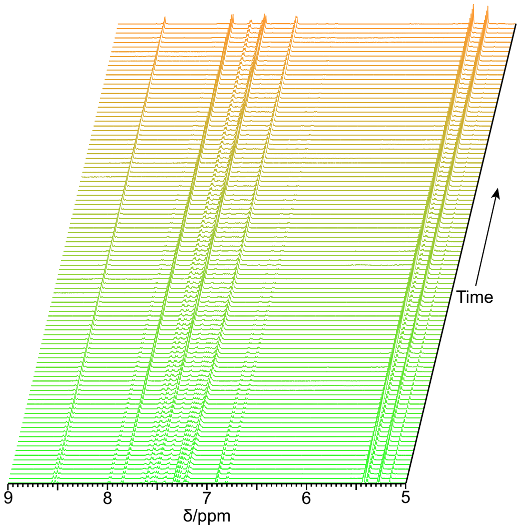

Figure S18. Temporal ^{1}H NMR thermal relaxation spectra (CD_3CN, 500 MHz, 318 K) Z-predominant **oAzoBox**$^{4+}$, obtained by irradiation at 350 nm.

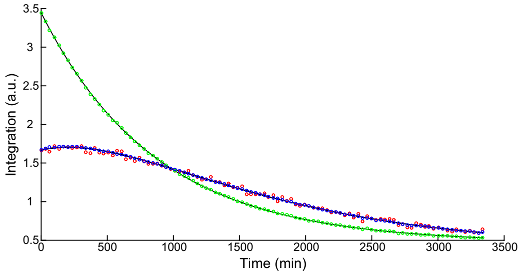

Figure S19. Kinetic fit of selected Hα proton resonances of the temporal ^{1}H NMR thermal relaxation spectra (CD_3CN, 500 MHz, 318 K) of Z-predominant **oAzoBox**$^{4+}$, obtained by irradiation at 350 nm. The integrations of the proton resonances were fitted to equations S6 and S7.

Table S2. Rate (κ, min^{-1}) and time constants (τ, min) for the rise and decay of the Hα proton resonances belonging to E,E-**oAzoBox**$^{4+}$, E,Z-**oAzoBox**$^{4+}$ and Z,Z-**oAzoBox**$^{4+}$ as measured by ^{1}H NMR (CD_3CN, 500 MHz, 318 K).

Proton	Shift [δ, ppm]	Rate Constant [κ, min^{-1}]	Error in κ [$\Delta\kappa$, min^{-1} x 10^{-6}]	Time Constant [τ, min]
E,Z-Hα	7.984	0.001057	13.1	945.64
Z,Z-Hα	6.904	0.00115^a	1.112	1564.95
Z,E-Hα	6.798	0.001051	2.49	951.31

^{a} Fit of the total integration area of the Z,Z-Hα proton resonance, such that the obtained rate constant is twice that of a single **AB** unit ($2\kappa_1$).

323 K

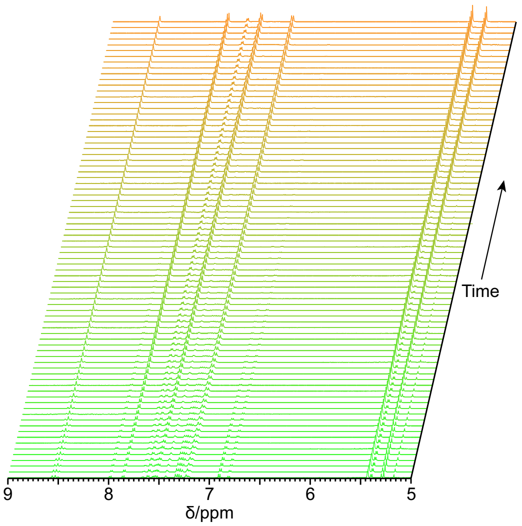

Figure S20. Temporal ^{1}H NMR thermal relaxation spectra (CD_3CN, 500 MHz, 323 K) of Z-predominant **oAzoBox**$^{4+}$, obtained by irradiation at 350 nm.

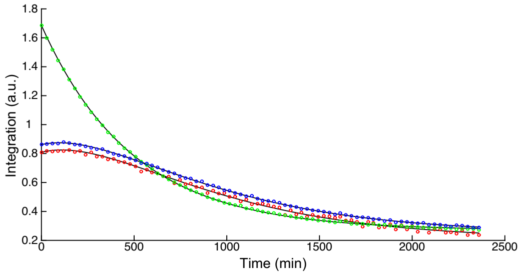

Figure S21. Kinetic fit of selected proton resonances of the temporal ^{1}H NMR thermal relaxation spectra (CD_3CN, 500 MHz, 323 K) of Z-predominant **oAzoBox**$^{4+}$, obtained by irradiation at 350 nm. The integrations of the proton resonances were fitted to equations S6 and S7.

Table S3. Rate (κ, min^{-1}) and time constants (τ, min) for the rise and decay of the Hα proton resonances belonging to E,E-**oAzoBox**$^{4+}$, E,Z-**oAzoBox**$^{4+}$ and Z,Z-**oAzoBox**$^{4+}$ as measured by ^{1}H NMR (CD_3CN, 500 MHz, 323 K).

Proton	Shift [δ, ppm]	Rate Constant [κ, min^{-1}]	Error in κ [$\Delta\kappa$, min^{-1} x 10^{-6}]	Time Constant [τ, min]
E,Z-Hα	7.984	0.001871	21.6	534.41
Z,Z-Hα	6.904	0.00201[a]	3.826	497.51
Z,E-Hα	6.798	0.001897	7.51	527.12

[a] Fit of the total integration area of the Z,Z-Hα proton resonance, such that the obtained rate constant is twice that of a single **AB** unit ($2\kappa_1$).

328 K

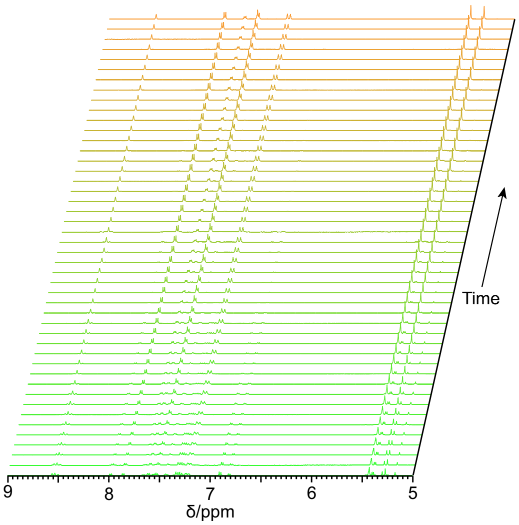

Figure S22. Temporal ^{1}H NMR thermal relaxation spectra (CD_3CN, 500 MHz, 328 K) of Z-predominant **oAzoBox**$^{4+}$, obtained by irradiation at 350 nm.

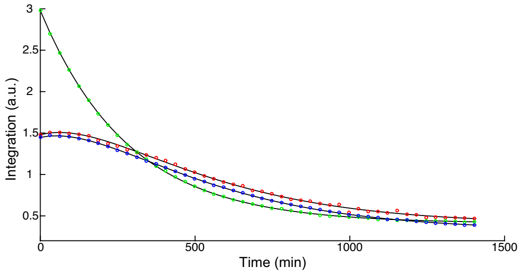

Figure S23. Kinetic fit of selected Hα proton resonances of the temporal ^{1}H NMR thermal relaxation spectra (CD_3CN, 500 MHz, 328 K) of Z-predominant **oAzoBox**$^{4+}$, obtained by irradiation at 350 nm. The integrations of the proton resonances were fitted to equations S6 and S7.

Table S4. Rate (κ, min^{-1}) and time constants (τ, min) for the rise and decay of the Hα proton resonances belonging to E,E-**oAzoBox**$^{4+}$, E,Z-**oAzoBox**$^{4+}$ and Z,Z-**oAzoBox**$^{4+}$ as measured by ^{1}H NMR (CD_3CN, 500 MHz, 328 K).

Proton	Shift [δ, ppm]	Rate Constant [κ, min^{-1}]	Error in κ [$\Delta\kappa$, min^{-1} x 10^{-6}]	Time Constant [τ, min]
E,Z-Hα	7.984	0.003294	31.1	303.56
Z,Z-Hα	6.904	0.00350[a]	7.017	285.71
Z,E-Hα	6.798	0.003303	9.78	302.72

[a] Fit of the total integration area of the Z,Z-Hα proton resonance, such that the obtained rate constant is twice that of a single **AB** unit ($2\kappa_1$).

Erying Plot (κ_1)

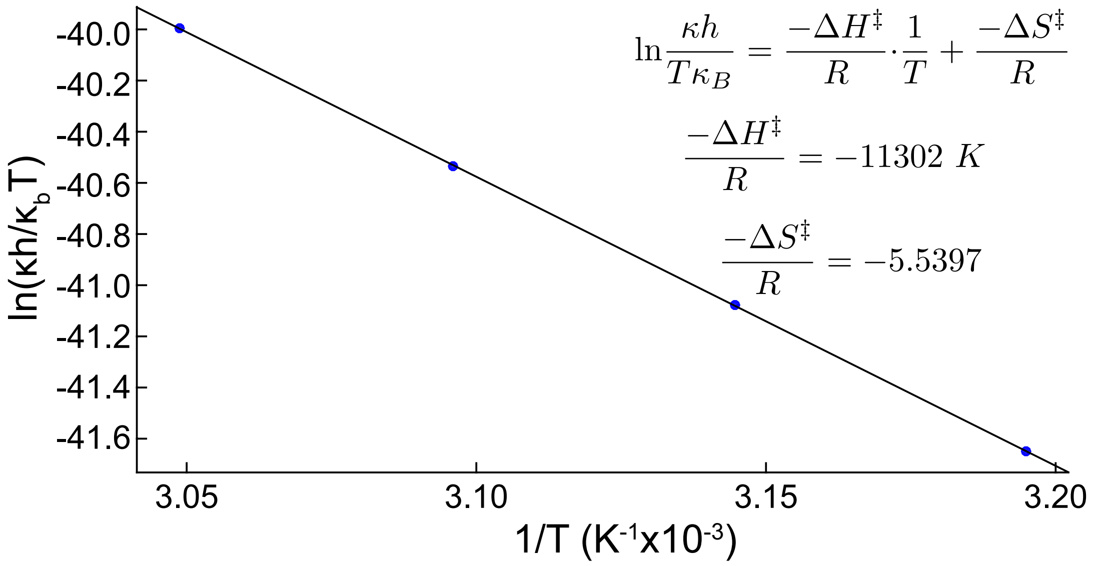

Figure S24. Erying plot for the rate constants (κ_1) of $Z{\rightarrow}E$ thermal isomerisation of *Z,Z*-**oAzoBox**$^{4+}{\rightarrow}$*E,Z*-**oAzoBox**$^{4+}$ measured at 313, 318, 323 and 328 K. The four values of κ_1 were obtained as half the values of the rate constants for the decay of the *Z,Z*-Hα resonance (Tables S1-S4). Inset: Fitted equation with the values of the slope and intercept.

Erying Plot (κ_2)

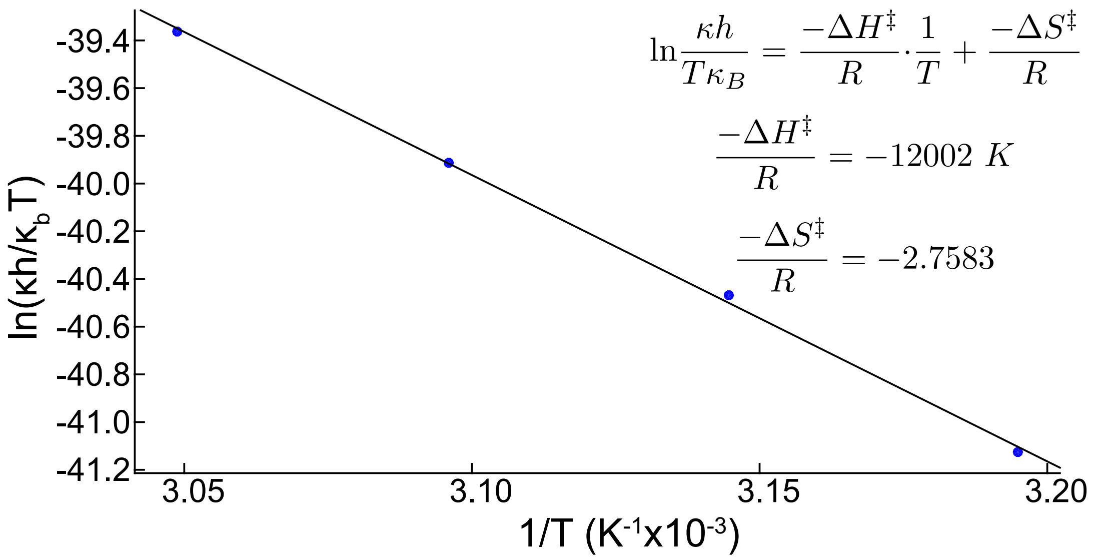

Figure S25. Erying plot for the rate constants (κ_2) of $Z{\rightarrow}E$ thermal isomerisation of *E,Z*-**oAzoBox**$^{4+}{\rightarrow}$*E,E*-**oAzoBox**$^{4+}$ measured at 313, 318, 323 and 328 K. The four values of κ_2 were obtained as the average values of the rate constants for the decays of the *E,Z*-Hα and *Z,E*-Hα resonances (Tables S1-S4). Inset: Fitted equation with the values of the slope and intercept.

5.1.2 $AzoBI^{2+}\cdot 2PF_6^-$

313 K

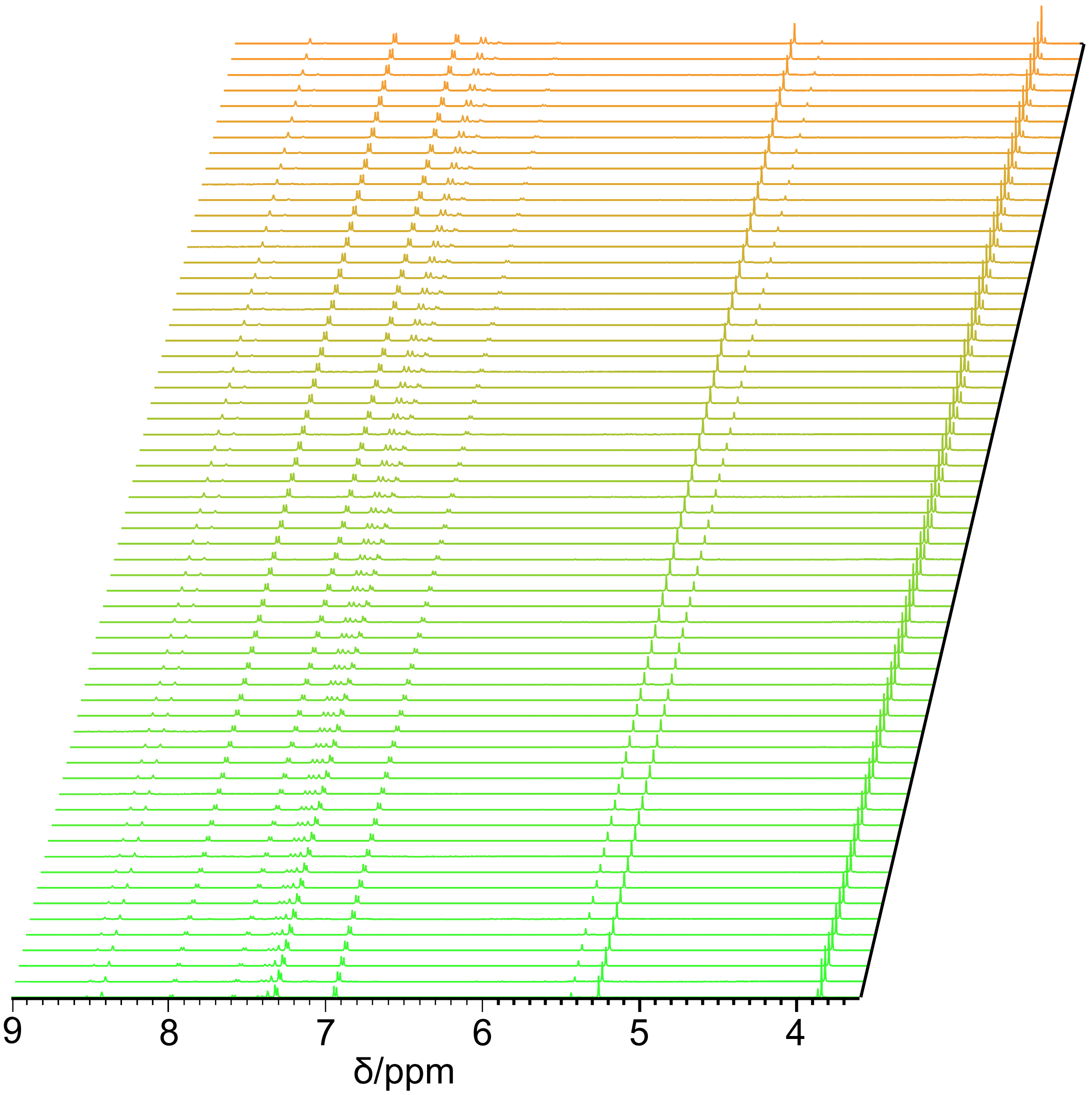

Figure S26. Temporal ^1H NMR thermal relaxation spectra (CD_3CN, 500 MHz, 313 K) of Z-predominant $\mathbf{AzoBI^{2+}}$, obtained by irradiation at 350 nm.

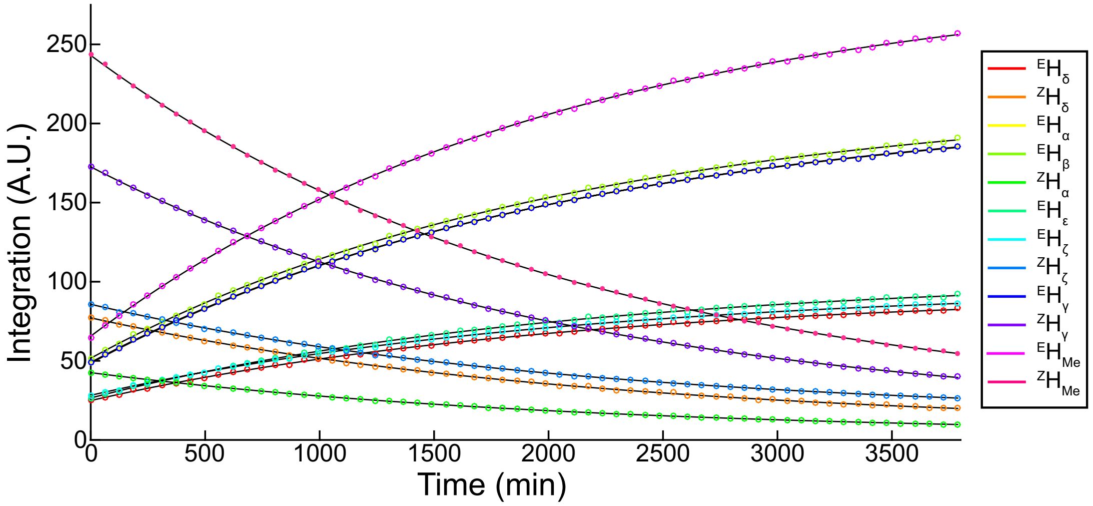

Figure S27. Kinetic fit of selected proton resonances of the temporal ^{1}H NMR thermal relaxation spectra (CD_3CN, 500 MHz, 313 K) of Z-predominant **AzoBI**$^{2+}$, obtained by irradiation at 350 nm. The integrations of the proton resonance were fitted to single time dependent exponential functions using DynamicsCentre2.3 software.

Table S5. Rate (κ, min^{-1}) and time constants (τ, min) for the rise and decay of the proton resonances belonging to E-**AzoBI**$^{2+}$ and Z-**AzoBI**$^{2+}$ as measured by ^{1}H NMR (CD_3CN, 500 MHz, 313 K)

Proton	Shift [δ, ppm]	Rate Constant [κ, min^{-1}]	Error in κ [$\Delta\kappa$, min^{-1} x 10^{-6}]	Time Constant [τ, min]
E-Hδ	8.519	0.000483	1.341	2070.39
Z-Hδ	8.422	0.000461	1.281	2169.20
E-Hα	7.986	0.000471	0.675	2123.14
E-Hβ	7.587	0.000477	0.670	2096.44
E-Hϵ	6.942	0.000486	1.091	2057.61
E-Hζ	7.436	0.000480	1.130	2083.33
Z-Hζ	7.405	0.000474	1.206	2109.70
Z-Hα	7.372	0.000477	0.459	2096.44
E-Hγ	5.444	0.000473	0.582	2114.16
Z-Hγ	5.268	0.000470	0.600	2127.66
E-H$_{Me}$	3.878	0.000477	0.344	2096.44
Z-H$_{Me}$	3.851	0.000477	0.361	2096.44
Z-**AzoBI**$^{2+}$	–	0.000476[a]	6.225[b]	2103.41 ± 27.76[c]

[a] Calculated from the average of all fitted resonances of Z-**AzoBI**$^{2+}$. [b] Calculated from the standard deviation of the rate constant (κ) for Z-**AzoBI**$^{2+}$. [c] Average time constant calculated from the average of all time constants (τ) and error calculated from the standard deviation of the rate constant for Z-**AzoBI**$^{2+}$.

318 K

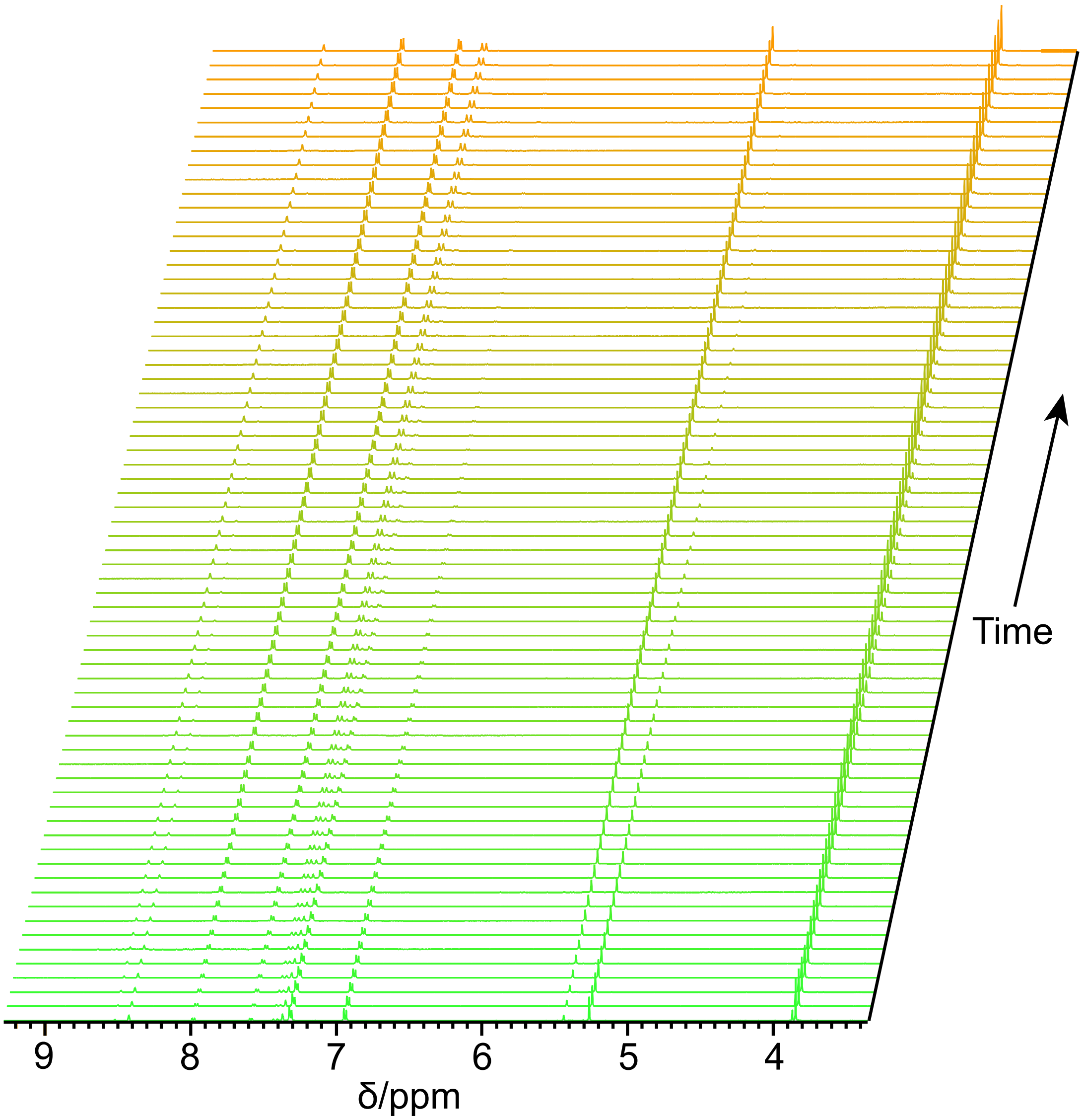

Figure S28. Temporal ^{1}H NMR thermal relaxation spectra (CD_3CN, 500 MHz, 318 K) of Z-predominant **AzoBI**$^{2+}$, obtained by irradiation at 350 nm.

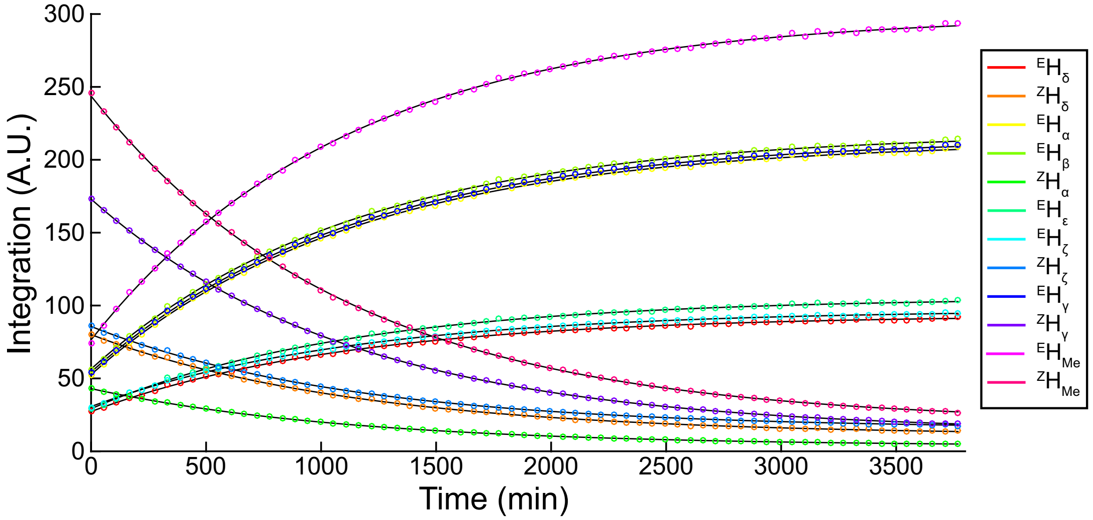

Figure S29. Kinetic fit of selected proton resonances of the temporal ^{1}H NMR thermal relaxation spectra (CD_3CN, 500 MHz, 318 K) of Z-predominant **AzoBI**$^{2+}$, obtained by irradiation at 350 nm. The integrations of the proton resonance were fitted to single time dependent exponential functions using DynamicsCentre2.3 software.

Table S6. Rate (κ, min^{-1}) and time constants (τ, min) for the rise and decay of the proton resonances belonging to E-**AzoBI**$^{2+}$ and Z-**AzoBI**$^{2+}$ as measured by ^{1}H NMR (CD_3CN, 500 MHz, 318 K)

Proton	Shift [δ, ppm]	Rate Constant [κ, min^{-1}]	Error in κ [$\Delta\kappa$, min^{-1} x 10^{-6}]	Time Constant [τ, min]
E-Hδ	8.519	0.000888	1.382	1126.13
Z-Hδ	8.422	0.000868	1.332	1152.07
E-Hα	7.986	0.000879	0.714	1137.66
E-Hβ	7.587	0.000880	0.662	1136.36
E-Hϵ	6.942	0.000873	1.142	1145.48
E-Hζ	7.436	0.000885	1.328	1129.94
Z-Hζ	7.405	0.000887	1.255	1127.40
Z-Hα	7.372	0.000875	0.488	1142.86
E-Hγ	5.444	0.000876	0.720	1141.55
Z-Hγ	5.268	0.000882	0.630	1133.79
E-H$_{Me}$	3.878	0.000897	0.413	1114.83
Z-H$_{Me}$	3.851	0.000896	0.409	1116.07
Z-**AzoBI**$^{2+}$	–	0.000882[a]	8.49[b]	1133.68 ± 10.89[c]

[a] Calculated from the average of all fitted resonances of Z-**AzoBI**$^{2+}$. [b] Calculated from the standard deviation of the rate constant (κ) for Z-**AzoBI**$^{2+}$. [c] Average time constant calculated from the average of all time constants (τ) and error calculated from the standard deviation of the rate constant for Z-**AzoBI**$^{2+}$.

323 K

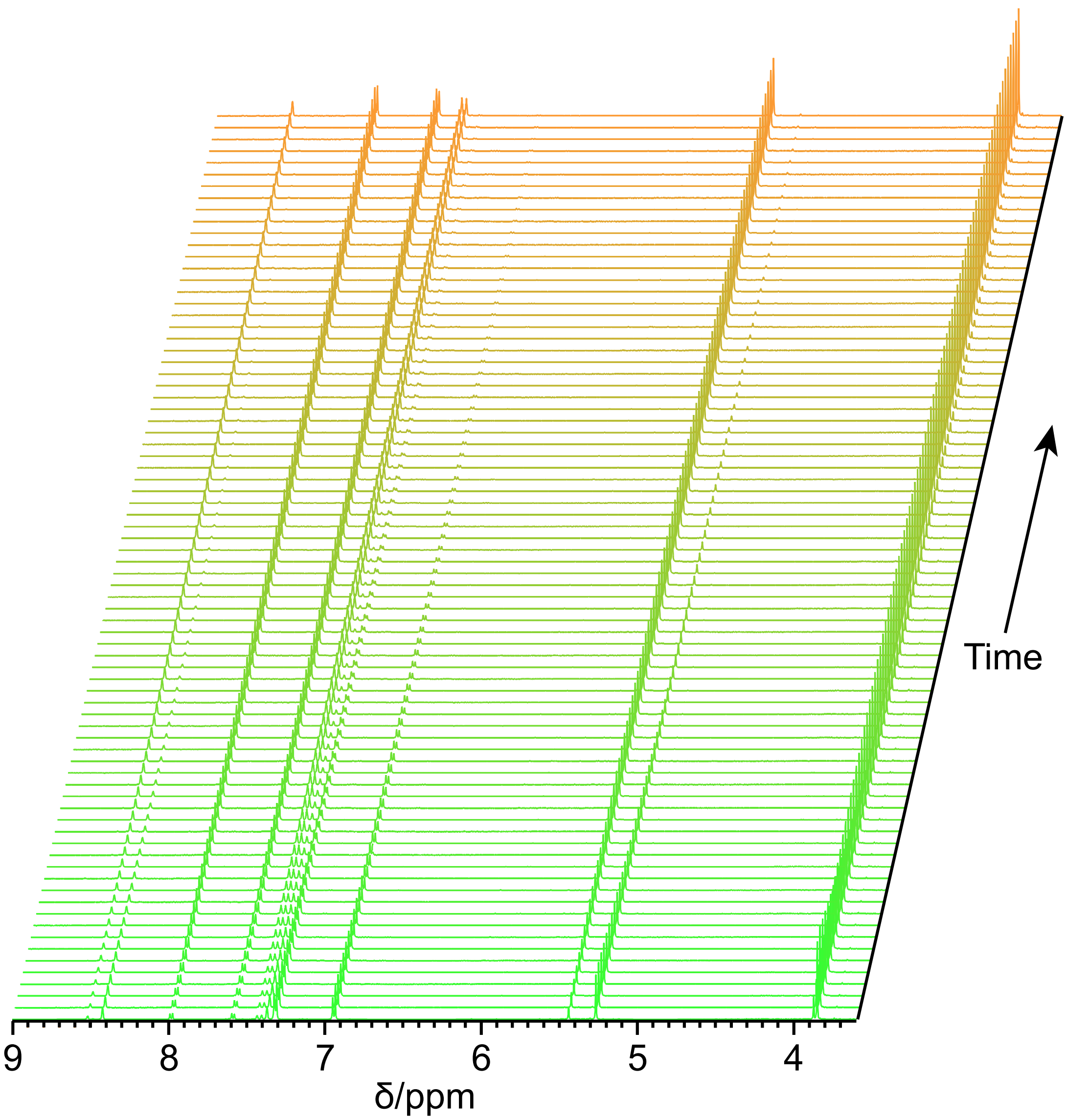

Figure S30. Temporal ^{1}H NMR thermal relaxation spectra (CD_3CN, 500 MHz, 323 K) of *Z*-predominant **AzoBI**$^{2+}$, obtained by irradiation at 350 nm.

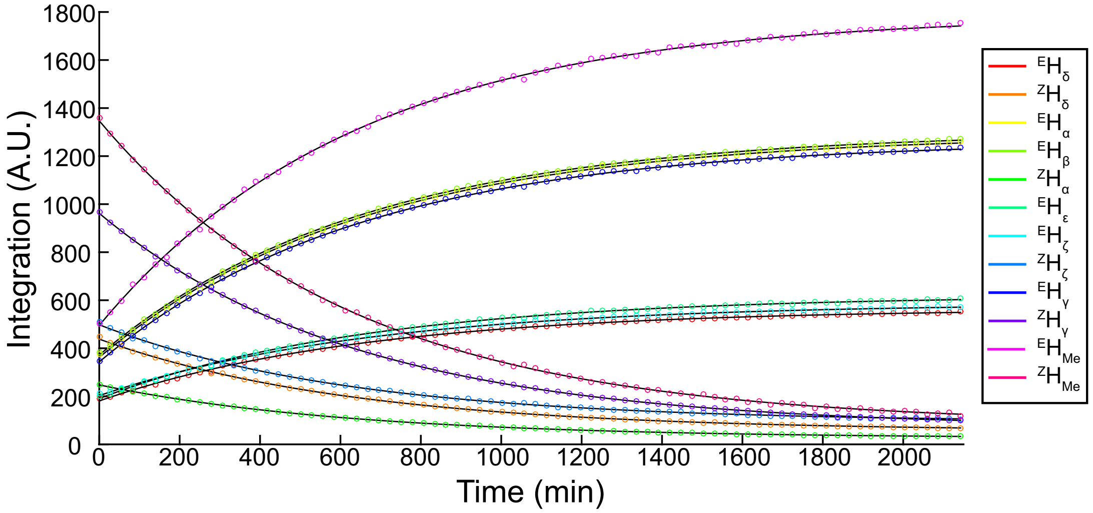

Figure S31. Kinetic fit of selected proton resonances of the temporal ^{1}H NMR thermal relaxation spectra (CD_3CN, 500 MHz, 323 K) of Z-predominant **AzoBI**$^{2+}$, obtained by irradiation at 350 nm. The integrations of the proton resonance were fitted to single time dependent exponential functions using DynamicsCentre2.3 software.

Table S7. Rate (κ, min^{-1}) and time constants (τ, min) for the rise and decay of the proton resonances belonging to E-**AzoBI**$^{2+}$ and Z-**AzoBI**$^{2+}$ as measured by ^{1}H NMR (CD_3CN, 500 MHz, 323 K)

Proton	Shift [δ, ppm]	Rate Constant [κ, min^{-1}]	Error in κ [$\Delta\kappa$, min^{-1} x 10^{-6}]	Time Constant [τ, min]
E-Hδ	8.519	0.00151	3.469	662.25
Z-Hδ	8.422	0.00157	3.288	636.94
E-Hα	7.986	0.00152	1.779	657.89
E-Hβ	7.587	0.00151	1.694	662.25
E-Hϵ	7.436	0.00149	2.774	671.14
E-Hζ	7.405	0.00149	3.013	671.14
Z-Hζ	7.372	0.00161	3.164	621.11
Z-Hα	6.942	0.00157	1.881	636.94
E-Hγ	5.444	0.00153	1.408	653.59
Z-Hγ	5.268	0.00157	1.357	636.94
E-H$_{Me}$	3.878	0.00156	0.9895	641.03
Z-H$_{Me}$	3.851	0.00158	0.8289	632.91
Z-**AzoBI**$^{2+}$	–	0.00154[a]	37.4[b]	648.68 ± 15.724[c]

[a] Calculated from the average of all fitted resonances of Z-**AzoBI**$^{2+}$. [b] Calculated from the standard deviation of the decay rate constant for Z-**AzoBI**$^{2+}$. [c] Average time constant calculated from the average of all time constants (τ) and error calculated from the standard deviation of the rate constant for Z-**AzoBI**$^{2+}$.

328 K

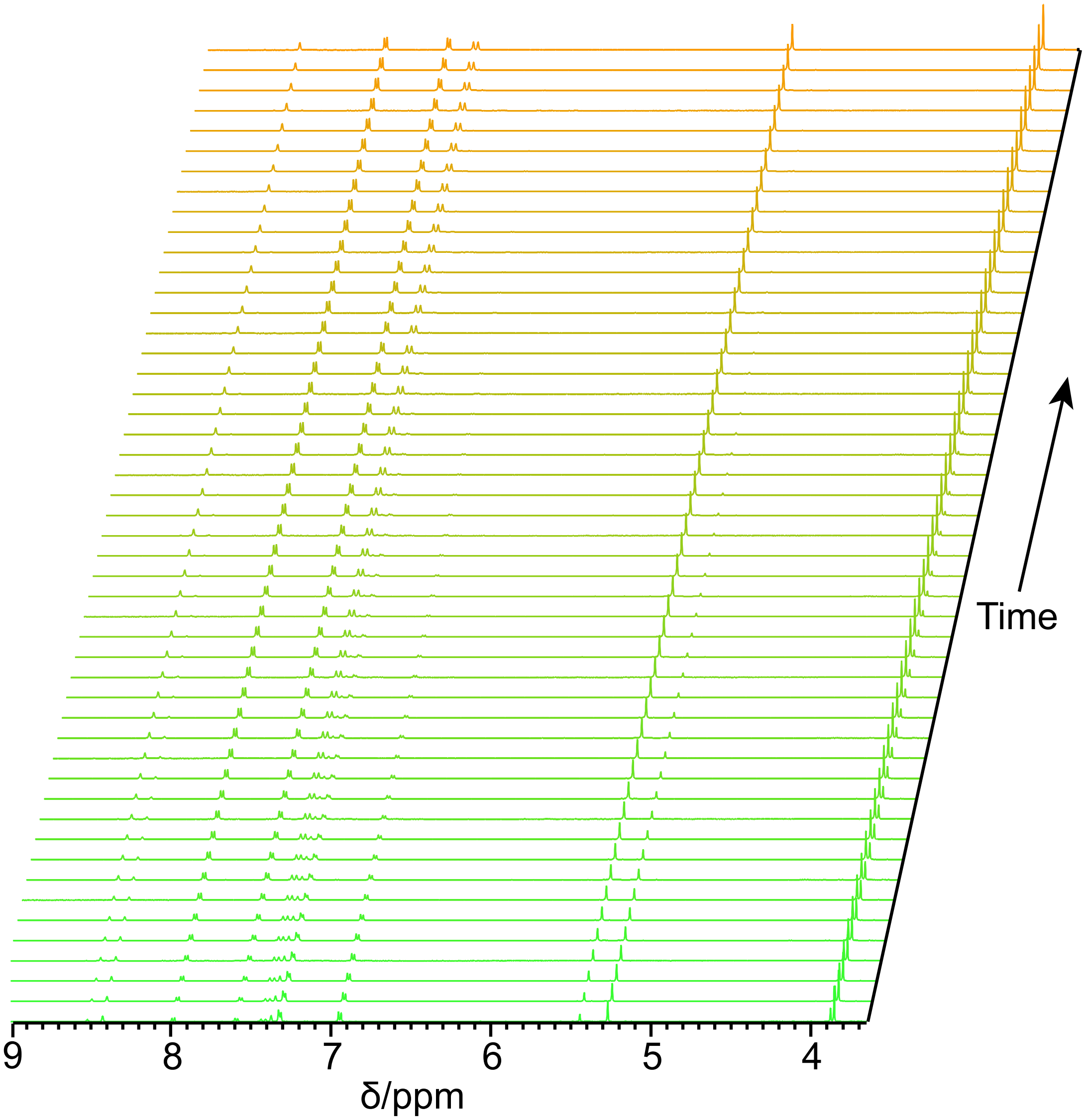

Figure S32. Temporal ^{1}H NMR thermal relaxation spectra (CD_3CN, 500 MHz, 328 K) of Z-predominant **AzoBI^{2+}**, obtained by irradiation at 350 nm.

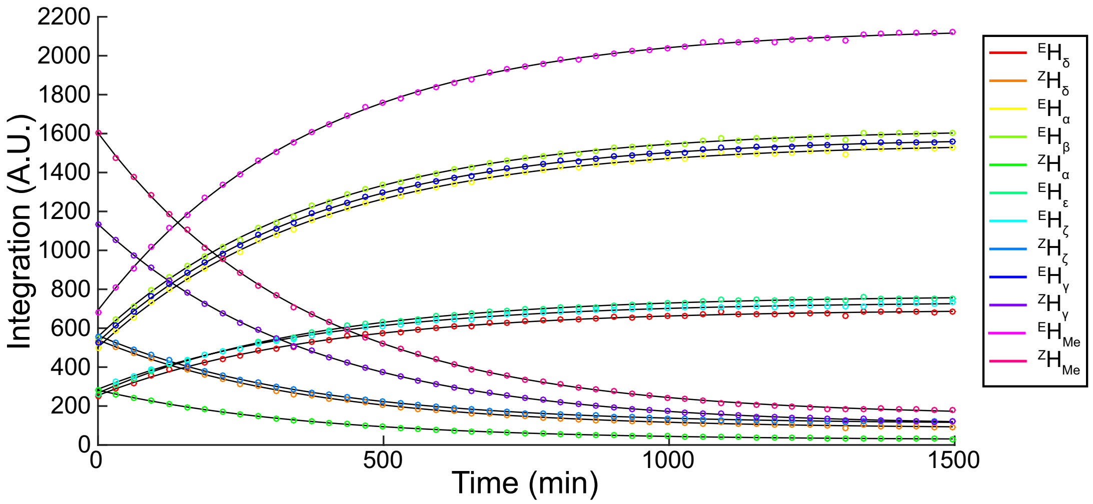

Figure S33. Kinetic fit of selected proton resonances of the temporal ^{1}H NMR thermal relaxation spectra (CD_3CN, 500 MHz, 328 K) of Z-predominant **AzoBI**$^{2+}$, obtained by irradiation at 350 nm. The integrations of the proton resonance were fitted to single time dependent exponential functions using DynamicsCentre2.3 software.

Table S8. Rate (κ, min^{-1}) and time constants (τ, min) for the rise and decay of the proton resonances belonging to E-**AzoBI**$^{2+}$ and Z-**AzoBI**$^{2+}$ as measured by ^{1}H NMR (CD_3CN, 500 MHz, 328 K)

Proton	Shift [δ, ppm]	Rate Constant [κ, min^{-1}]	Error in κ [$\Delta\kappa$, min^{-1} x 10^{-6}]	Time Constant [τ, min]
E-Hδ	8.519	0.00260	7.612	384.62
Z-Hδ	8.422	0.00267	8.016	374.53
E-Hα	7.986	0.00260	3.857	384.62
E-Hβ	7.587	0.00261	4.096	383.14
E-Hϵ	6.942	0.00260	6.138	384.62
E-Hζ	7.436	0.00262	6.763	381.68
Z-Hζ	7.405	0.00276	6.654	362.32
Z-Hα	7.372	0.00266	3.907	375.94
E-Hγ	5.444	0.00261	3.669	383.14
Z-Hγ	5.268	0.00268	3.320	373.13
E-H$_{Me}$	3.878	0.00266	2.064	375.94
Z-H$_{Me}$	3.851	0.00273	2.072	366.30
Z-**AzoBI**$^{2+}$	–	0.00265[a]	51.316[b]	377.50 ± 7.2[c]

[a] Calculated from the average of all fitted resonances of Z-**AzoBI**$^{2+}$. [b] Calculated from the standard deviation of the rate constant (κ) for Z-**AzoBI**$^{2+}$. [c] Average time constant calculated from the average of all time constants (τ) and error calculated from the standard deviation of the rate constant for Z-**AzoBI**$^{2+}$.

Erying Plot

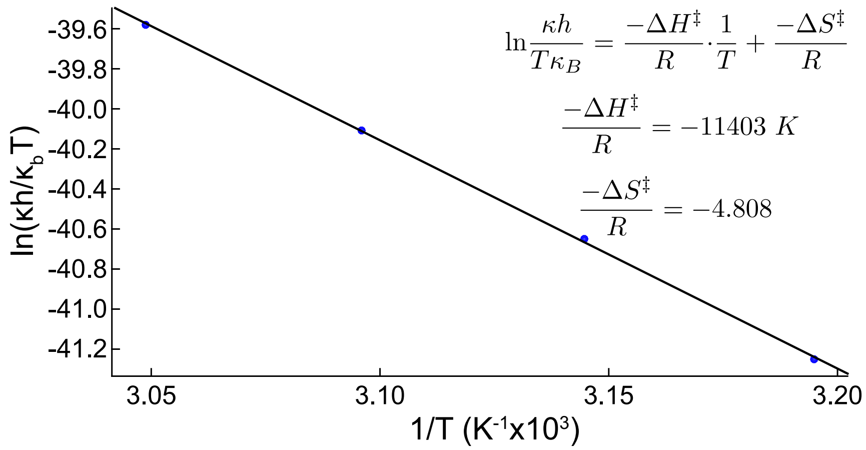

Figure S34. Erying plot for the rate constants of $Z\rightarrow E$ thermal isomerisation of Z-**AzoBI**$^{2+}$$\rightarrow$$E$-**AzoBI**$^{2+}$ measured at 313, 318, 323 and 328 K. The four values of κ were obtained as the average values of the moduli of the rate constants for the rise or decay of all resonances (Tables S5-S8). Inset: Fitted equation with the values of the slope and intercept.

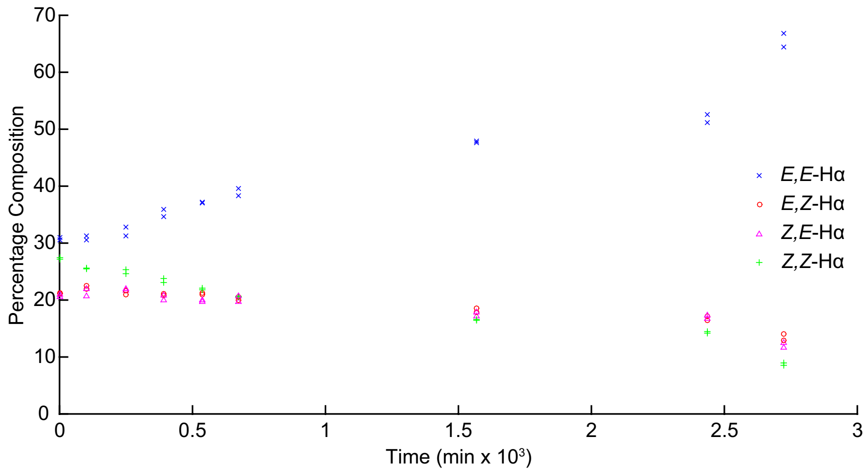

Figure S35. Plot of the percentage composition, monitored by ^{1}H NMR (Hα resonances) over time during the thermal $Z\rightarrow E$ isomerisation of Z-predominant **oAzoBox**$^{4+}$ in the presence and absence of **4DPDO**. The samples were kept at identical temperatures throughout the approximate three week time span of the experiment, which were monitored as fluctuating between 14°C and 20°C.

5.2 Additional Guests for oAzoBox^{4+}

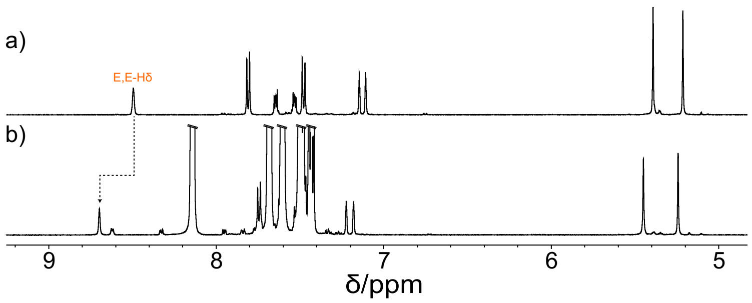

Figure S36. ^{1}H NMR spectra (CD_3CN, 500 MHz) of (a) **oAzoBox^{4+}** and (b) **oAzoBox^{4+}** and excess **PPO**.

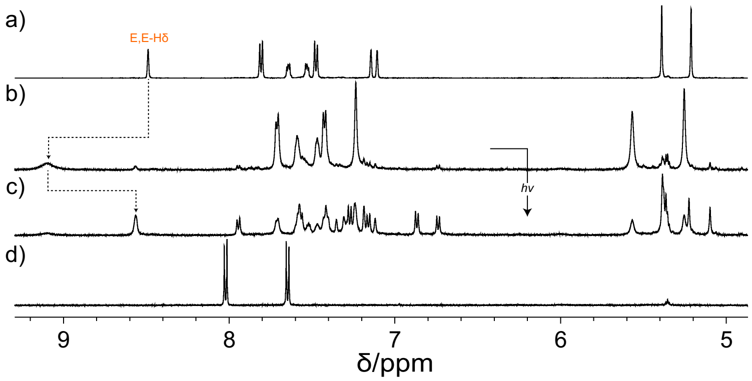

Figure S37. ^{1}H NMR spectra (CD_3CN, 500 MHz) of (a) **oAzoBox^{4+}**, (b) **oAzoBox^{4+}** and excess **BPDC** (c) **oAzoBox^{4+}** and excess **BPDC** after exposure to UV light (350 nm) and (d) **BPDC**.

6 Electrospray Ionisation Mass Spectrometry

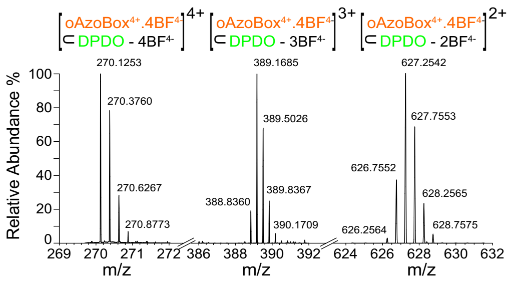

Figure S38. Partial ESI-MS spectrum of E,E-**oAzoBox^{4+}⊂4DPDO** (CH_3CN).

7 X-ray Crystallography

7.1 oAzoBox^{4+}·4BF$_4$$^-$

7.1.1 Crystallization Methods

X-Ray quality crystals of **oAzoBox^{4+}·4BF$_4$$^-$** were grown by the slow diffusion of *i*-Pr$_2$O vapours into an MeCN solution of **oAzoBox^{4+}·4BF$_4$$^-$**. Crystals formed under ambient conditions at room temperature over a period of days and were seen to be single and free of defects by use of an optical microscope fitted with a crossed-polarizer. Crystals were removed from the mother liquor and protected from desolvation by submersion in paratone oil before being mounted using an appropriate MiTeGen tip and flash frozen under a continuous stream of N_2.

7.1.2 X-ray Crystallography

(a) oAzoBox^{4+}·4BF$_4$$^-$: Data were collected at 180 K on a Bruker D8-QUEST diffractometer equipped with an Incoatec IμS Cu microsource ($\lambda = 1.5418$ Å) and PHOTON-100 CMOS detector. The data were collected and processed using APEX2, and a multi-scan correction was applied using SADABS[5].

All crystallographic data are available free of charge from the Cambridge Crystallographic Data Centre (CCDC) *via* www.ccdc.cam.ac.uk/data_request/cif.

7.1.3 Crystallographic Data

The crystallographic information, structural parameters and additional refinement details for **oAzoBox^{4+}·4BF$_4$$^-$** is given below.

(a) **oAzoBox^{4+}·4BF$_4$$^-$**: $C_{68}H_{64}B_4F_{16}N_{16}$; orange block, 0.260 x 0.120 x 0.080 mm^3; triclinic, space group $P2_1/c$; $a = 20.5508(7)$, $b = 9.5608(3)$, $c = 19.40296(6)$ Å; $\alpha = 90$, $\beta = 116.996(2)$, $\gamma = 90°$; $V = 3396.9(2)$ Å^3; $Z = 2$; $\rho_{calcd} = 1.373$ Mg m^{-3}; $2\theta_{max} = 100.864°$; $T = 180(2)$ K; 19951 reflections collected, 3350 independent. $\mu = 0.983$ mm^{-1}; $R_{int} = 0.0312$; $R_1 = 0.0800$ [$I > 2.0\sigma$(I)], $wR_2 = 0.2636$ (all data); CCDC deposition number **1497573**.

8 Computational Studies

8.1 Energy Minimised Structures of oAzoBox^{4+}

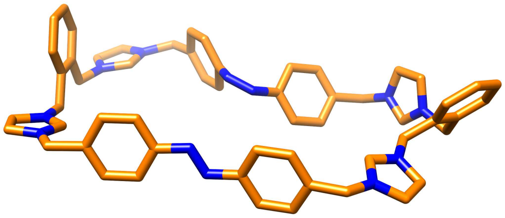

Figure S39. Geometry-optimised molecular structure (B3LYP-D3(BJ)/TZVP level of theory) of *E,Z*-**oAzoBox**$^{4+}$.

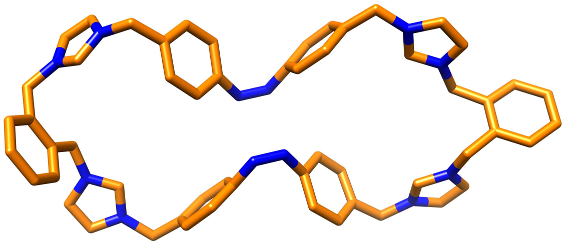

Figure S40. Geometry-optimised molecular structure (B3LYP-D3(BJ)/TZVP level of theory) of *Z,Z*-**oAzoBox**$^{4+}$.

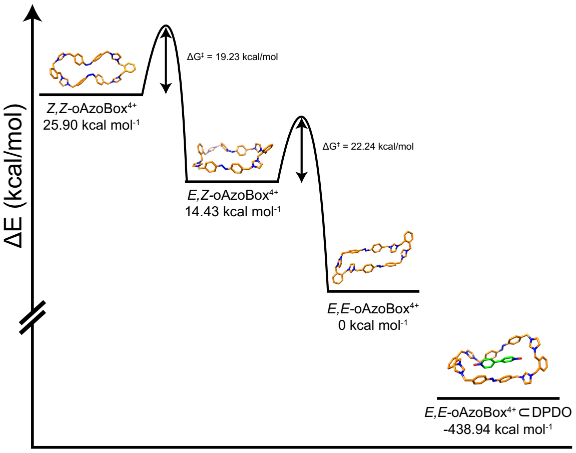

Figure S41. Structures, relative energies and thermal $Z \rightarrow E$ isomerisation activation energies of the three stereoisomers of **oAzoBox**$^{4+}$ and *E,E*-**oAzoBox**$^{4+}$⊂**4DPDO**. The energy is measured in kcal mol^{-1} and are compared to the lowest energy conformation of *E,E*-**oAzoBox**$^{4+}$, which is set to 0 kcal mol^{-1}. The energy of *E,E*-**oAzoBox**$^{4+}$⊂**4DPDO** was calculated by taking the ground state energy of uncomplexed **4DPDO** into account.

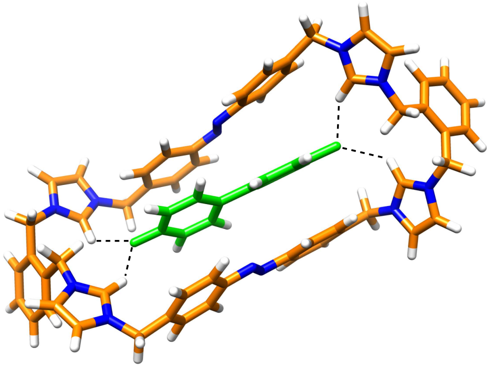

Figure S42. Geometry-optimised molecular structure (B3LYP-D3(BJ)/TZVP level of theory) of *E,E*-**oAzoBox**$^{4+}$$\subset$**4DPDO** revealing hydrogen bonding (dashed lines) between the Hδ resonances of *E,E*-**oAzoBox**$^{4+}$ and the oxygen atoms of **4DPDO**.

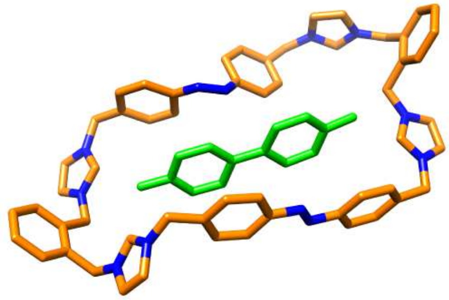

Figure S43. Geometry-optimised molecular structure (B3LYP-D3(BJ)/TZVP level of theory) of *E,Z*-**oAzoBox**$^{4+}$$\subset$**4DPDO**.

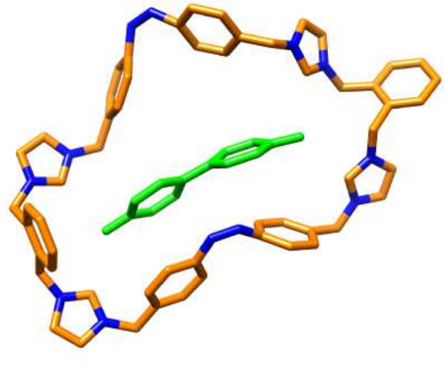

Figure S44. Geometry-optimised molecular structure (B3LYP-D3(BJ)/TZVP level of theory) of *Z,Z*-**oAzoBox**$^{4+}$$\subset$**4DPDO**.

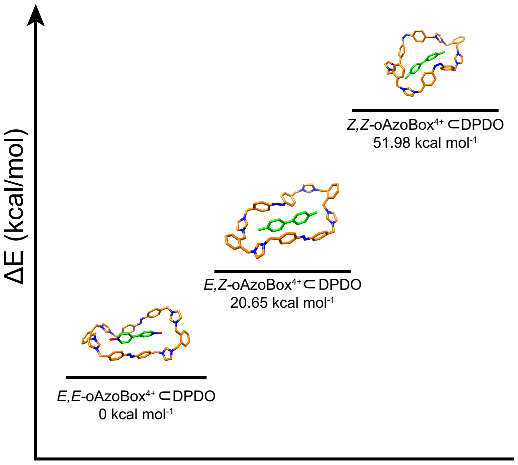

Figure S45. Structures, relative energies and thermal $Z \rightarrow E$ isomerisation activation energies of the three stereoisomers of **oAzoBox**$^{4+}$ and *E,E*-**oAzoBox**$^{4+}$⊂**4DPDO**. The energy is measured in kcal mol^{-1} and are compared to the lowest energy conformation of *E,E*-**oAzoBox**$^{4+}$, which is set to 0 kcal mol^{-1}. The energy of *E,E*-**oAzoBox**$^{4+}$⊂**4DPDO** was calculated by taking the ground state energy of uncomplexed **4DPDO** into account.

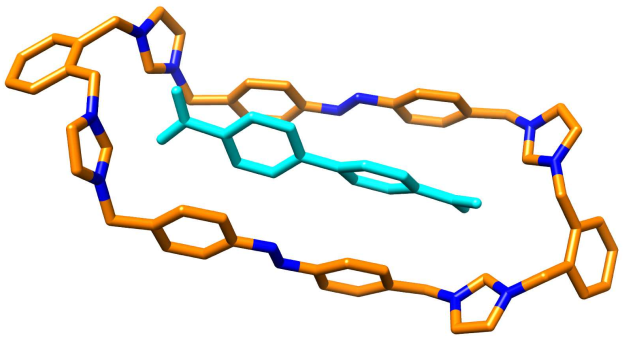

Figure S46. Geometry-optimised molecular structure (B3LYP-D3(BJ)/TZVP level of theory) of *E,E*-**oAzoBox**$^{4+}$⊂**BPDC**.

8.2 Relaxed Potention Energy Surface Scans for Thermal $Z \rightarrow E$ Isomerisation of oAzoBox^{4+}

Table S9. Tabulated data for the relaxed potential energy surface scan around the C-N=N-C- azo torsion angle from *Z,Z*-**oAzoBox**$^{4+}$ to *E,Z*-**oAzoBox**$^{4+}$.

Torsion Angle	Energy (a.u.)	Energy (kcal mol^{-1})	ΔE (kcal mol^{-1})
13	-2822.17396	-1770922.62	0.000
43	-2822.166459	-1770917.92	4.702
73	-2822.143842	-1770903.73	18.90
103	-2822.137727	-1770899.89	22.73
133	-2822.172238	-1770921.55	1.08
163	-2822.191908	-1770933.89	-11.26
193	-2822.187292	-1770930.99	-8.37

Table S10. Tabulated data for the relaxed potential energy surface scan around the C-N=N-C- azo torsion angle from *E,Z*-**oAzoBox**$^{4+}$ to *E,E*-**oAzoBox**$^{4+}$.

Torsion Angle	Energy (a.u.)	Energy (kcal mol^{-1})	ΔE (kcal mol^{-1})
12	-2822.19222	-1770934.08	0.00
42	-2822.18502	-1770929.57	4.52
72	-2822.16408	-1770916.43	17.66
102	-2822.15748	-1770912.29	21.80
132	-2822.19621	-1770936.59	-2.50
162	-2822.21995	-1770951.49	-17.40
192	-2822.219099	-1770950.95	-16.87

8.3 Energy Minimised Structures of oAzoBI^{2+}

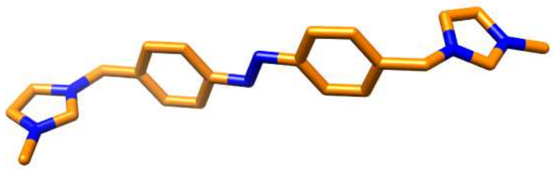

Figure S47. Geometry-optimised molecular structure (B3LYP-D3(BJ)/TZVP level of theory) of E-**AzoBI**$^{2+}$.

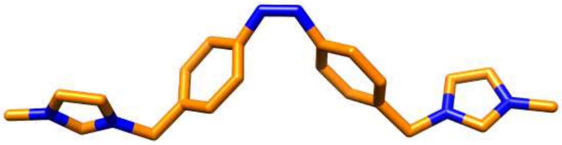

Figure S48. Geometry-optimised molecular structure (B3LYP-D3/TZV level of theory) of Z-**AzoBI**$^{2+}$.

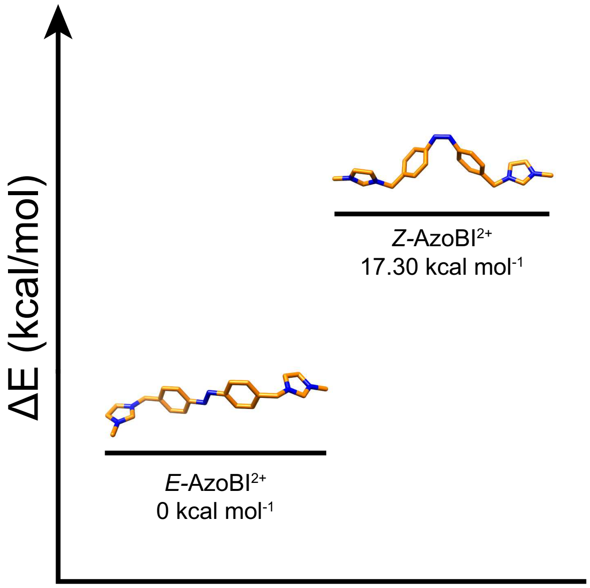

Figure S49. Structures and relative energies the two stereoisomers of **oAzoBI**$^{2+}$. The energy is measured in kcal mol^{-1} and are compared to the lowest energy conformation of E-**oAzoBI**$^{I+}$, which is set to 0 kcal mol^{-1}.

9 Phototriggered Guest Release

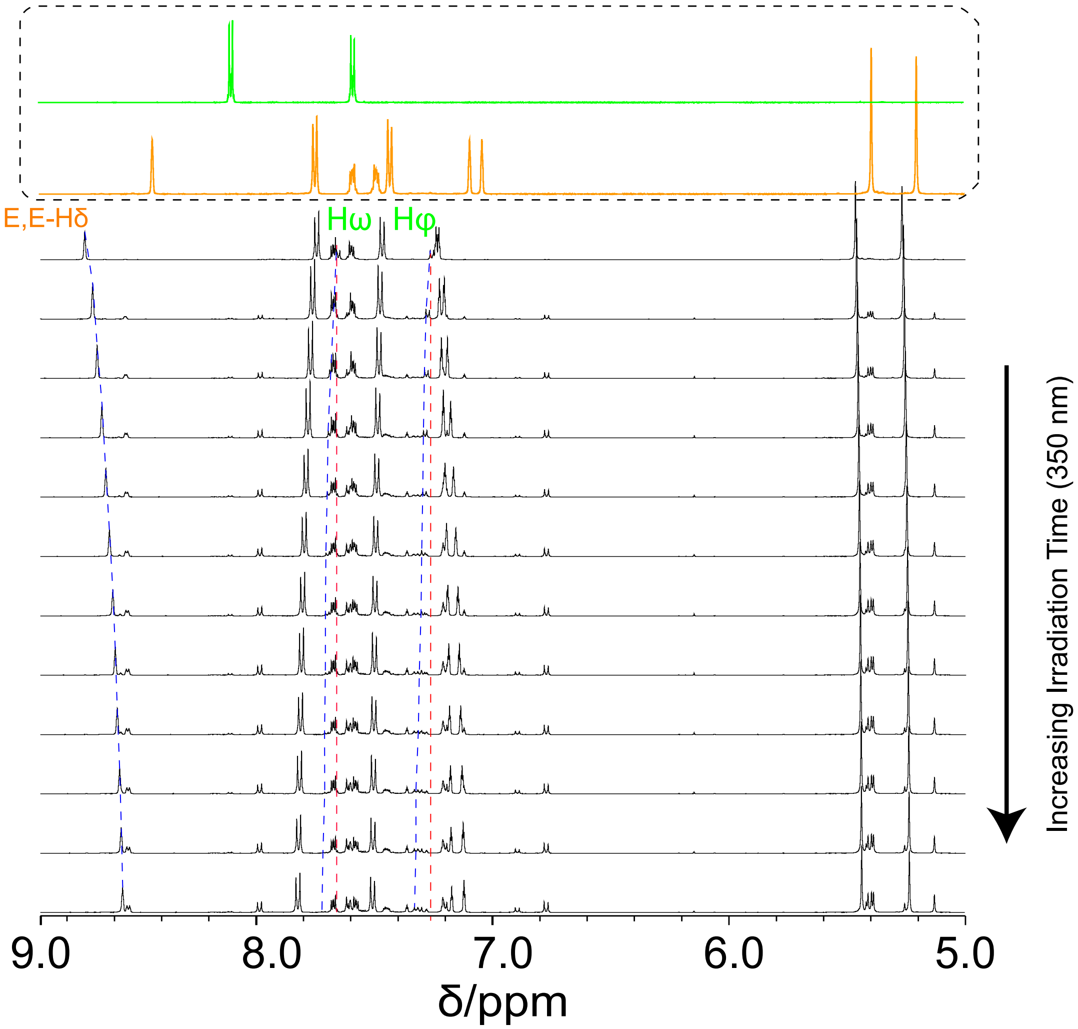

Figure S50. ^{1}H NMR spectra (CD_3CN, 500 MHz) of *E,E*-**oAzoBox**$^{4+}$⊂**4DPDO** upon increasingly longer exposure to UV light (350 nm). Blue dotted lines track the shifting proton resonances of **4DPDO** and *E,E*-H_δ and the red lines indicate the non-shifted **4DPDO** proton resonances. The upfield shift of the **4DPDO** protons indicates release from the *E,E*-**oAzoBox**$^{4+}$ cavity as a result of hydrogen bonding competition. The green and orange ^{1}H NMR spectra (CD_3CN, 500 MHz, top) are that of **4DPDO** and *E,E*-**oAzoBox**$^{4+}$, respectively.

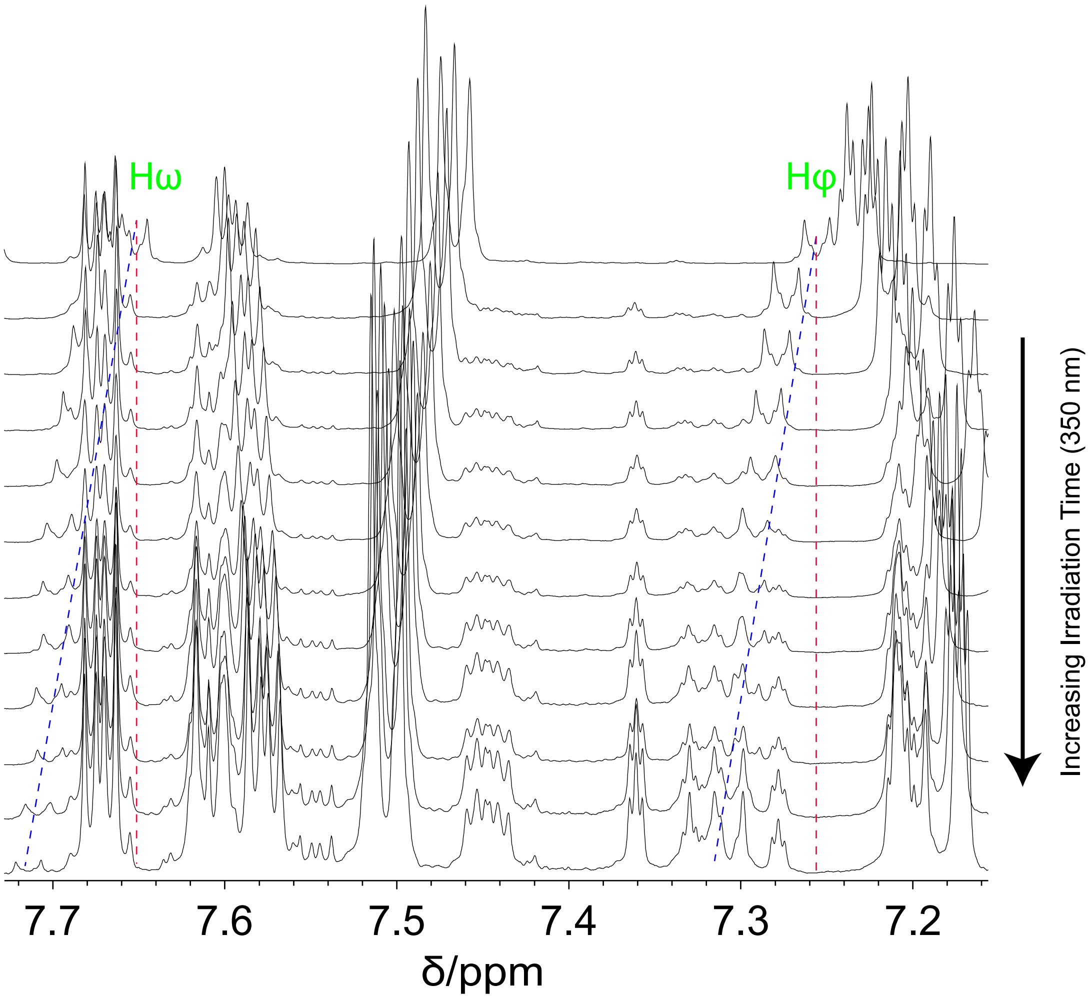

Figure S51. ^{1}H NMR spectra (CD_3CN, 500 MHz) of *E,E*-**oAzoBox**$^{4+}$$\subset$**4DPDO** upon increasingly longer exposure to UV light (350 nm, Zoom in of Figure S50). Blue dotted lines track the shifting proton resonances of **4DPDO** and the red lines indicate the non-shifted **4DPDO** proton resonances.

References

[1] Stappert, K., Muthmann, J., Spielberg, E. T. & Mudring, A.-V. Azobenzene-based organic salts with ionic liquid and liquid crystalline properties. *Crystal Growth & Design* **15**, 4701–4712 (2015).

[2] Gong, H.-Y., Rambo, B. M., Lynch, V. M., Keller, K. M. & Sessler, J. L. “Texas-sized” molecular boxes: Building blocks for the construction of anion-induced supramolecular species via self-assembly. *J. Am. Chem. Soc.* **135**, 6330–6337 (2013).

[3] Grimme, S., Ehrlich, S. & Goerigk, L. Effect of the damping function in dispersion corrected density functional theory. *J. Comp. Chem.* **32**, 1456–1465 (2011).

[4] Sure, R. & Grimme, S. Comprehensive benchmark of association (free) energies of realistic host-guest complexes. *J. Chem. Theory Comput.* **11**, 3785–3801 (2015).

[5] Bruker APEX2 and SADABS, Bruker AXS, Madison, Wisconsin, USA. (2014).
